# Gut microbiome stability and dynamics in healthy donors and patients with non-gastrointestinal cancers

**DOI:** 10.1084/jem.20200606

**Published:** 2020-11-11

**Authors:** Allyson L. Byrd, Menghan Liu, Kei E. Fujimura, Svetlana Lyalina, Deepti R. Nagarkar, Bruno Charbit, Jacob Bergstedt, Etienne Patin, Oliver J. Harrison, Lluís Quintana-Murci, Ira Mellman, Darragh Duffy, Matthew L. Albert, the Milieu Intérieur Consortium

**Affiliations:** 1 Department of Cancer Immunology, Genentech Inc., San Francisco, CA; 2 Sackler Institute of Graduate Biomedical Sciences, New York University School of Medicine, New York, NY; 3 Personalized Health Care, Genentech Inc., San Francisco, CA; 4 Cytometry and Biomarkers UTechS, CRT, Institut Pasteur, Paris, France; 5 Human Evolutionary Genetics Unit, Institut Pasteur, UMR 2000, Centre National de la Recherche Scientifique, Paris, France; 6 Center for Fundamental Immunology, Benaroya Research Institute, Seattle, WA; 7 Collège de France, Paris, France; 8 Translational Immunology Lab, Institut Pasteur, Paris, France; 9 Insitro, South San Francisco, CA

## Abstract

As microbial therapeutics are increasingly being tested in diverse patient populations, it is essential to understand the host and environmental factors influencing the microbiome. Through analysis of 1,359 gut microbiome samples from 946 healthy donors of the Milieu Intérieur cohort, we detail how microbiome composition is associated with host factors, lifestyle parameters, and disease states. Using a genome-based taxonomy, we found biological sex was the strongest driver of community composition. Additionally, bacterial populations shift across decades of life (age 20–69), with Bacteroidota species consistently increased with age while Actinobacteriota species, including *Bifidobacterium*, decreased. Longitudinal sampling revealed that short-term stability exceeds interindividual differences. By accounting for these factors, we defined global shifts in the microbiomes of patients with non-gastrointestinal tumors compared with healthy donors. Together, these results demonstrated that the microbiome displays predictable variations as a function of sex, age, and disease state. These variations must be considered when designing microbiome-targeted therapies or interpreting differences thought to be linked to pathophysiology or therapeutic response.

## Teaser

By characterizing gut microbiome composition across 946 healthy donors, Byrd et al. demonstrate strong influences of age and biological sex. Additionally, they define global shifts in the gut microbiome composition of patients with non-gastrointestinal tumors compared with healthy donors.

## Graphical Abstract

**Figure:**
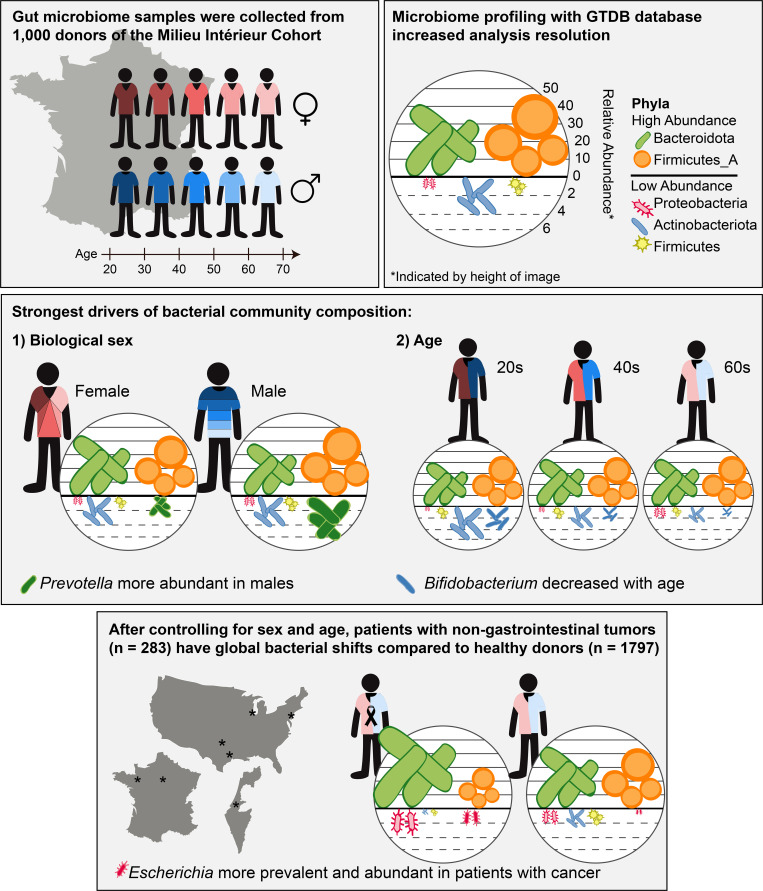

## Introduction

Microbial therapeutics, including fecal microbiota transplants (FMTs), bacterial consortia, and probiotics, are increasingly being tested in patients with *Clostridium difficile* infections and other gastrointestinal (GI) disorders (Allegretti et al., 2019), including inflammatory bowel disease (IBD) and, more recently, non-GI indications such as autism (Kang et al., 2019) and cancer (Mullard, 2018). In parallel to microbial therapeutics, microbial signatures are being evaluated as a novel class of biomarkers, applied for stratification of efficacy and safety in clinical trials across multiple indications (Ananthakrishnan et al., 2017; Dubin et al., 2016). Notably, this rapid increase in microbial therapeutics and biomarkers demands a rigorous reevaluation of the factors influencing an individual’s personal gut microbiome over time. Such understanding is essential for optimizing clinical trials with any microbial component. For example, without a complete understanding of the factors influencing the gut microbiome in health and disease, we cannot determine whether the optimal FMT should be sourced from a patient who previously responded to a therapy or a healthy donor who is matched for age and sex.

In this paper, we present a comprehensive assessment of the gut microbiome of 946 well-defined healthy French donors from the Milieu Intérieur (MI) Consortium, with 1,359 shotgun metagenomic samples. Designed to study the genetic and environmental factors underlying immunological variance between individuals, the MI Consortium comprises 500 women and 500 men evenly stratified across five decades of life, from 20 to 69 yr of age, for whom extensive metadata, including demographic variables, serological measures, dietary information, and systemic immune profiles, are available and easily accessible (Patin et al., 2018; Thomas et al., 2015). Integrating these data with those from cancer patients, we demonstrate clear evidence for altered microbial communities in cancer patients across multiple non-GI indications.

To build on the findings of several landmark microbiome studies (Falony et al., 2016; Human Microbiome Project Consortium, 2012; Zhernakova et al., 2016), many of which relied on an older reference library for taxonomic classification of microbial sequence reads (Truong et al., 2015), we leveraged an expanded set of reference genomes with a novel taxonomy that corrects many misclassifications in public databases to discover new biological insights, particularly around age and sex (Parks et al., 2018). Notably, an independent dataset was used for replication of many of the findings (Zeevi et al., 2015). Study of short-term longitudinal samplings from half the donors found that individuals are more similar to themselves over time compared with others (Costello et al., 2009; Flores et al., 2014; Mehta et al., 2018). However, the degree of stability between individuals was quite variable and was influenced by lifestyle factors as well as baseline composition.

Overall, the aims of the study are threefold. First, we introduce a new microbiome analysis approach that uses an expanded set of reference genomes with a novel taxonomy to discover new, statistically robust insights into host/bacteria biology that will enable personalized medicine approaches for microbial therapeutics and biomarkers. Second, we provide the rich metadata and 1,000-plus deep shotgun metagenomic samples described here as a resource on which future microbiome studies can test and build new computational tools, as well as be compared against disease cohorts. Finally, while demonstrating the utility of this resource as a control population, we define global shifts in the gut microbiomes of patients with non-GI tumors compared with healthy donors.

## Results

### The Genome Taxonomy Database (GTDB) improves taxonomic resolution of *k*-mer–based approaches

**Figure S1.**
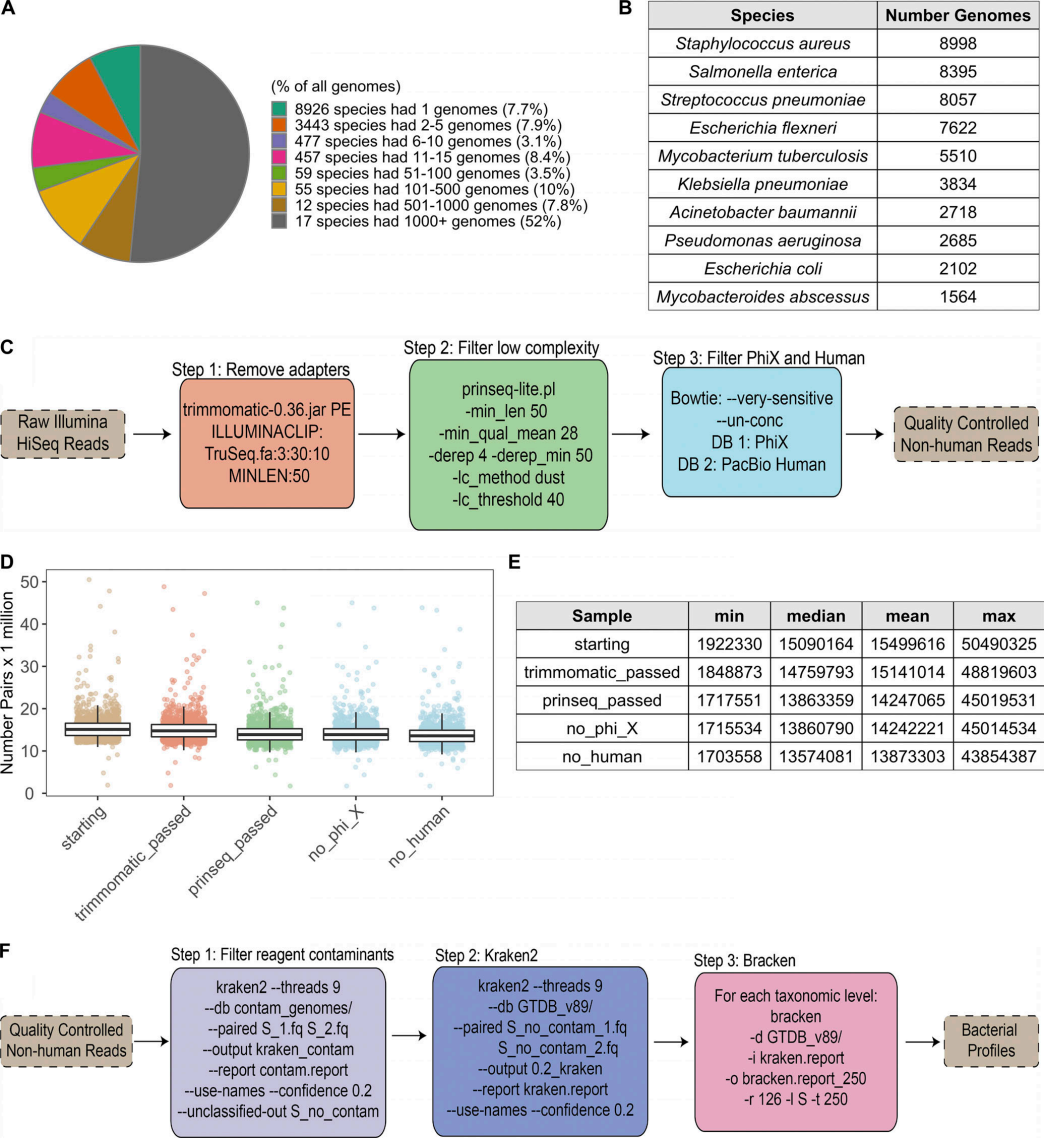
**Quality control and Kraken analysis pipeline.** Related to Fig. 1. **(A)** Pie chart indicating the distribution of genomes in RefSeq (as of June 25, 2019) to different species, based on GTDB taxonomy. 29 species represent >60% of the reference genomes. **(B)** Table of the 10 species with the greatest number of genomes in RefSeq. **(C)** Steps taken to quality control shotgun metagenomic reads. Additional details can be found in Materials and methods. **(D)** Bar plots show the number of paired-end reads remaining after each filtration step in C. Each point corresponds to one sample (*n* = 1,359). Colors correspond to steps in C. **(E)** Summary table for the number of paired-end reads in D. max, maximum; min, minimum. **(F)** Steps taken to run Kraken2 and Bracken2 on the quality-controlled reads, including removal of putative reagent contaminants. Additional details can be found in Materials and methods. See also Data S1, table 2.

Historically, microbial sequencing efforts focused predominantly on a small number of organisms, often causes of nosocomial infections (Fig. S1, A and B). By contrast, reference databases, including National Center for Biotechnology Information (NCBI) GenBank, are increasingly populated with genomic information of commensal microbes (Browne et al., 2016; Forster et al., 2019; Poyet et al., 2019; Zou et al., 2019). As genome reference databases expand, historical, microbiology-based taxonomic assignments do not reflect population-level relationships inferred from genome sequencing. This is particularly problematic for *k*-mer–based analyses, which use sequence similarity between closely related genomes to infer which taxa are present (Nasko et al., 2018).

To overcome these issues, in lieu of the traditional NCBI taxonomy, we generated a custom reference database of 23,505 RefSeq genomes with GTDB taxonomies (see Materials and methods and Data S1, table 1). Briefly, GTDB is a bacterial taxonomy based on a concatenated protein phylogeny in which polyphyletic groups were removed and taxonomic ranks were normalized on the basis of relative evolutionary divergence (Parks et al., 2018). The impact of this procedure was particularly prominent for species of the genus *Clostridium*, which were split into 121 unique genera spanning 29 families (Parks et al., 2018). This could be especially meaningful for analysis of gut microbiome samples, as *Clostridium* species are prevalent community members and often emerge in association studies.

**Figure S2.**
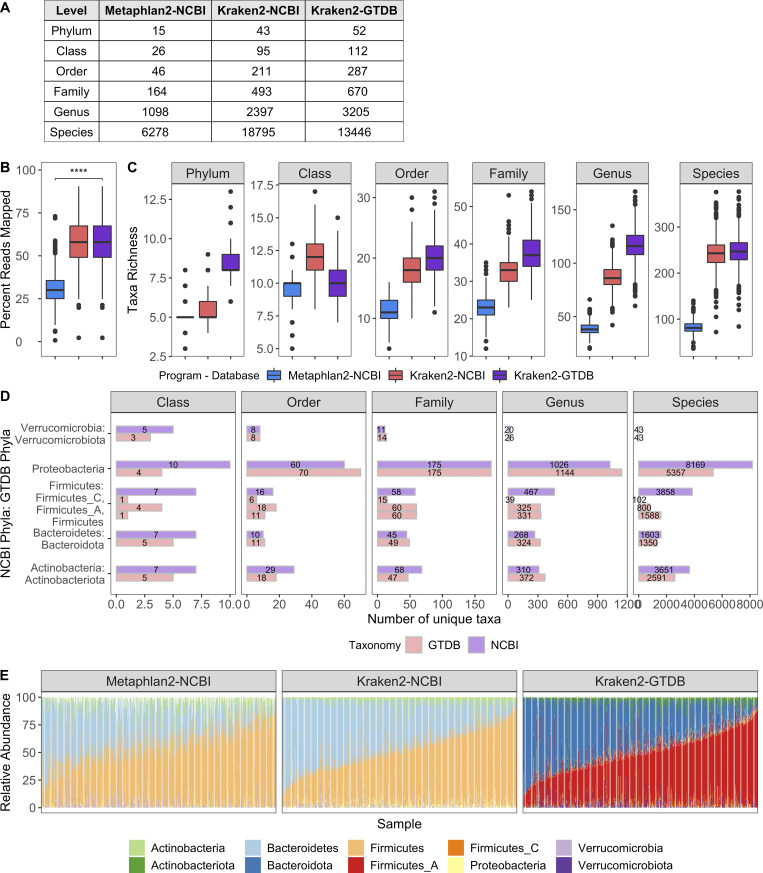
**Comparison of all program–database combinations tested.** Related to Fig. 1.** (A)** Table indicates the richness across taxonomic levels of the different databases tested. The NCBI and GTDB databases used by Kraken2 include the same 23,505 RefSeq genomes with taxonomies assigned either by NCBI or the GTDB, respectively. GTDB taxonomic assignments often lead to collapsing or splitting of those taxa originally in NCBI; e.g., Firmicutes was split into Firmicutes, Firmicutes_A, Firmicutes_B, Firmicutes_C, etc. **(B)** Boxplots compare the percentage of shotgun metagenomic reads mapped by the different program–database combinations. Percentage of mapped reads for Metaphlan2 was based on its estimated number of reads from the clade; ****, P < 2.22 × 10^−16^ by Wilcoxon rank sum. **(C)** Boxplots compare the richness of taxa identified per sample with the different program–database combinations tested. All pairwise comparisons had P < 0.0001 by Wilcoxon rank sum. **(D)** Bars indicate the number of unique taxa present in the NCBI (purple) and GTDB (red) databases for each of the top phyla across taxonomic levels. **(E)** Relative abundance of the top phyla across different program–database combinations. Each bar corresponds to one sample. Samples are ordered based on the relative abundance of Firmicutes_A found by Kraken2-GTDB.

The RefSeq sequences and taxonomic tree from the GTDB, including its naming conventions, were used to build a reference database for the *k*-mer–based program Kraken2 (Wood and Salzberg, 2014) and read-reassignment step Bracken2 (Lu et al., 2017). This custom Kraken2/GTDB pipeline was applied to 1,359 quality-controlled samples from 946 MI donors (Fig. S1, C–F; Data S1, tables 2 and 3) and compared using both the marker gene–based tool Metaphlan2 (Truong et al., 2015) and Kraken2, with the same 23,505 reference genomes using their original NCBI taxonomies (Fig. S2). Consistently, more bacterial taxa were identified per sample with Kraken2 than Metaphlan2, a result of the updated reference database and higher sensitivity of this *k*-mer–based approach (Fig. S2, A–C; McIntyre et al., 2017). Between the two Kraken databases (GTDB and NCBI), richness varied depending on how taxa were redistributed by GTDB. For example, GTDB split 2,397 NCBI genera into 3,205, while it collapsed 18,795 NCBI species into 13,446 (Fig. S2, A and D). Despite finer-level differences, the overall distribution of phyla across the three approaches was similar (Fig. S2 E), indicating that Kraken2/GTDB pipeline results would be consistent with previous analyses. As such, a combination of *k*-mer–based read assignment and genome-based taxonomy allows higher-resolution analysis of shotgun metagenomic samples.

### Variable gut microbiomes in a restricted geographical region

**Figure 1.**
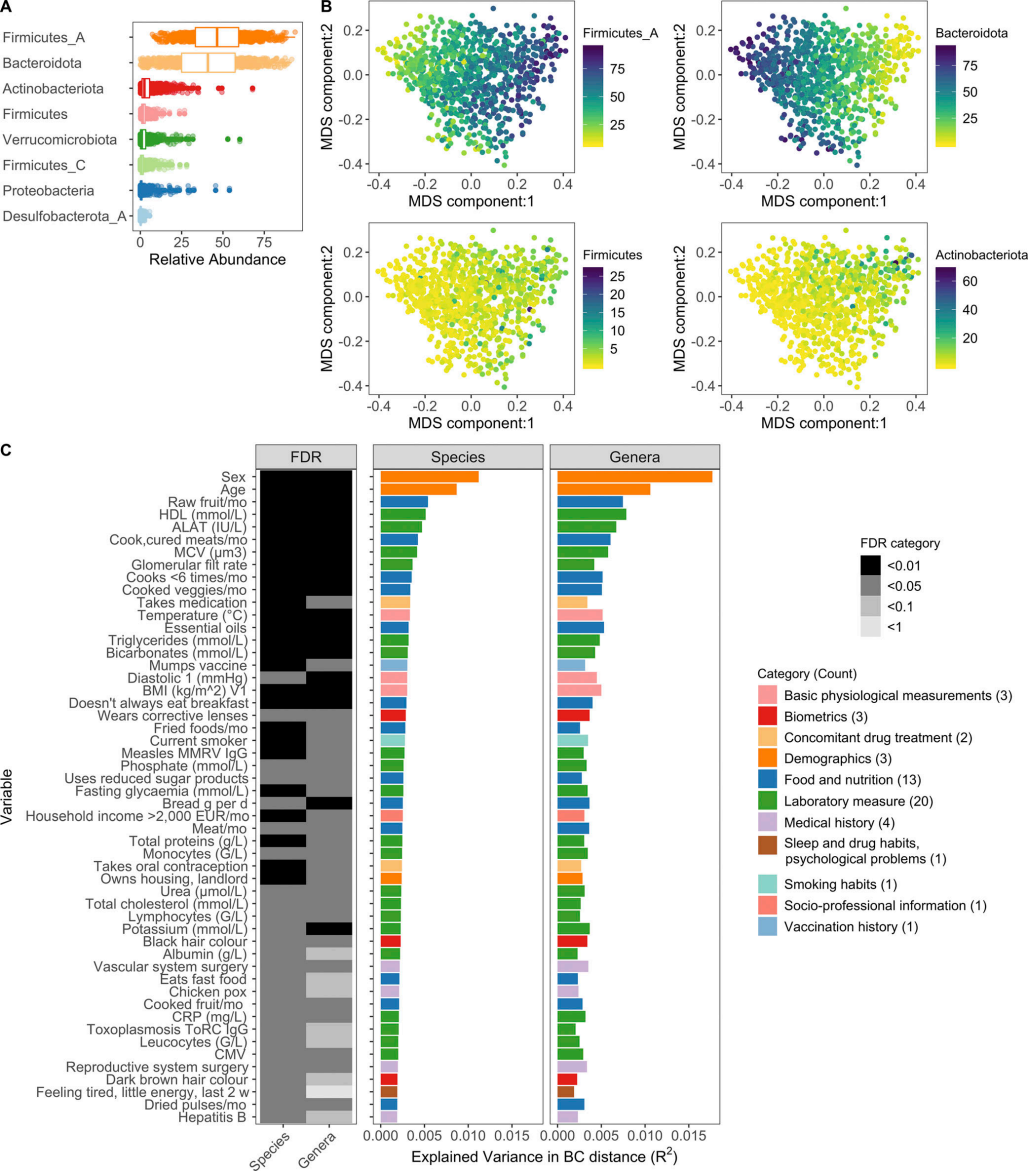
**Interindividual variation of bacterial composition is associated with many factors.** **(A)** Boxplots of the top eight phyla. Each dot corresponds to one donor. Firmicutes_A, Firmicutes_C, and Firmicutes were split into unique phyla by the GTDB. **(B)** MDS plots of BC distance of bacterial species composition. Ordination was primarily driven by the top two phyla Firmicutes_A and Bacteroidota. Each dot corresponds to one donor while color indicates relative abundance of each phyla. **(C)** In total, 52 factors (Benjamini–Hochberg FDR < 0.05) were associated with interindividual variation of the gut microbiome. The bar plots indicate the amount of interindividual variance explained by each factor for the species and genera level BC distance. Variables are ordered by the percentage variance explained in Kraken species. Colors of the bars correspond to the broad metadata category. The rectangles to the left indicate the statistical strength, as measured by FDR, of the association. For each variable, samples with NA values were excluded. For all these analyses, one sample per donor was used: when available, V1; if not V2. See also Fig. S1, Fig. S2, Fig. S3, and Data S1, tables 1–7. CMV, cytomegalovirus; CRP, C-reactive protein; EUR, Euro; HDL, high-density lipoprotein; MCV, mean corpuscular volume; MMRV, measles, mumps, rubella, and varicella vaccine.

To complement our optimized taxa-based approach and further use the resolution afforded by shotgun metagenomic sequencing, we applied HUMANn2 to identify the functional potential of microbial pathways present in the MI samples (Franzosa et al., 2018; Data S1, table 4). Using both the Kraken2/GTDB and HUMANn2 pipelines, we identified a broad range of diversity across the 946 individuals in this geographically restricted cohort of healthy French adults. This diversity was observed in terms of metabolic pathway richness (282 ± 40, mean ± SD), species richness (248 ± 32), and Shannon diversity (3.7 ± 0.35), which accounts for both richness and evenness (Data S1, table 2). Across donors, our GTDB pipeline confirmed Firmicutes and Bacteroidota (formerly Bacteroidetes) as the most abundant phyla in the gut, but enabled distinction among the original Firmicutes phyla, which was further divided in the GTDB into 12 distinct categories: Firmicutes, Firmicutes_A, Firmicutes_B, … Firmicutes_K (Data S1, table 1). Notably, throughout the GTDB, the group containing type material (if known) kept the original unsuffixed name. Of those, seven were present in this cohort, with Firmicutes_A the most abundant, followed by Firmicutes and Firmicutes_C (Fig. 1 A and Data S1, table 3), highlighting the finer granularity, even at the phylum level, provided by GTDB-based taxonomic calls. Subsequent application of the Bray–Curtis (BC) distance metric, a means to assess species presence/absence in addition to relative abundance across donors, demonstrated that samples fell along a gradient defined by the relative abundances of Firmicutes_A and Bacteroidota, with lesser contributions from Actinobacteriota and Firmicutes (first dimension of multidimensional scaling [MDS] projection; Fig. 1 B).

**Figure S3.**
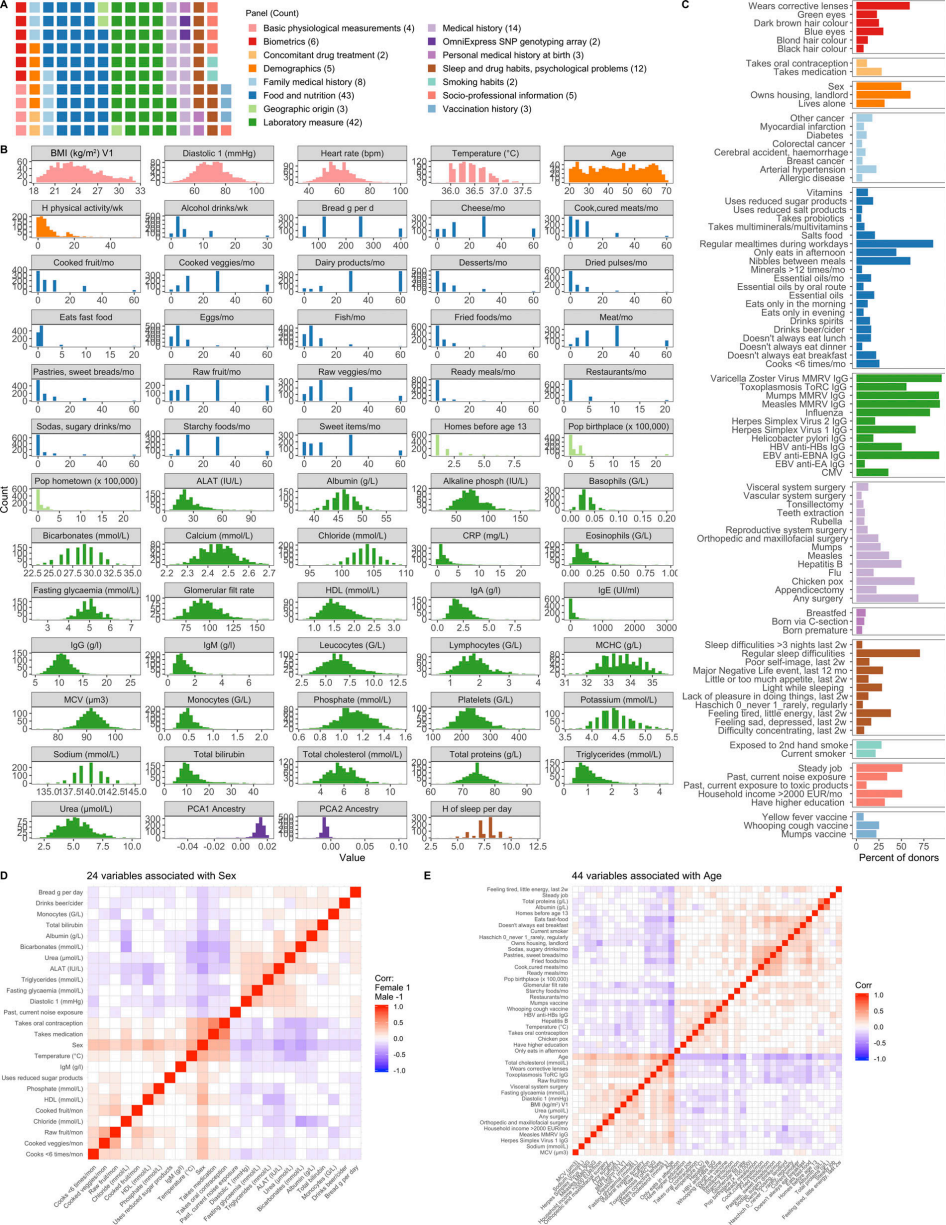
**154 variables were associated with bacterial profiles.** Related to Fig. 1. **(A)** Distribution of variables across broad categories. **(B)** Distribution of 64 continuous variables across donors. Colors correspond to those in A. **(C)** Percentage of donors for whom each of the 90 binary variables was true. Colors correspond to those in A. 2w, 2 wk; G/L, billion cells per liter; HBV, hepatitis B virus; MCHC, mean corpuscular hemoglobin concentration; mon, month; PC, principal component of genetics SNP array. **(D)** 24 variables were associated with sex with Spearman ρ > 0.2 or less than −0.2. Corr, correlation. **(E)** 44 variables were associated with age with Spearman ρ > 0.2 or less than −0.2. See also Data S1, tables 5 and 6. CMV, cytomegalovirus; CRP, C-reactive protein; EUR, Euro; HDL, high density lipoprotein; MCV, mean corpuscular volume; MMRV, measles, mumps, rubella, and varicella vaccine; PCA, principal component ancestry.

Using this extensively characterized cohort, we explored how 154 metadata variables (Fig. S3, A–C; and Data S1, tables 5 and 6), including 42 laboratory measurements and 43 dietary variables, contributed to overall bacterial community composition. We identified 52 variables (34% of total) that associated with Kraken2-GTDB species (permutational multivariate ANOVA [PERMANOVA] test false discovery rate [FDR] < 0.05); 51 of which replicated at the genera level (PERMANOVA test FDR < 0.1; Fig. 1 C and Data S1, table 7). The top contributors were age and sex, with lesser contributions from diet, such as consumption of raw fruit and cooked and cured meats, as well as frequency of fast food consumption, in line with previous reports of 16S rRNA analyses from this cohort (Partula et al., 2019; Scepanovic et al., 2019). Notably, sex and age were associated with 24 and 44 of the other metadata variables, respectively, which confounds our ability to dissociate the individual effects of these variables on microbial community composition (Fig. S3, D and E). In total, these factors explained <10% of population variability, indicating that the majority of variance in community composition remains unexplained. Drawbacks of this analysis are the absence of Bristol stool score, a measure of stool consistency, and levels of chromogranin A, a protein secreted by enteroendocrine cells, the factors most associated with community composition in previous European cohorts (Falony et al., 2016; Zhernakova et al., 2016). Although genetic data were also available for these donors, they were not considered here based on previous analyses that the effects of host genetics on microbiome are minimal in this (Scepanovic et al., 2019) and other (Rothschild et al., 2018) cohorts, in part owing to the small population sizes by genome-wide association study standards (Goodrich et al., 2017).

In this healthy cohort, medication usage was low, with only 28% of individuals (*n* = 266) taking medication of any kind. Notably, donors were excluded if they used antibiotics in the 3 mo preceding enrollment. Of all medications, only oral contraception was taken by >10% of participants (*n* = 111). In premenopausal women, oral contraception was taken by 36% (110/303) and explained 0.005% of the variance (P = 0.04). In contrast, relatively common medications, β-blockers and proton pump inhibitors, were taken by only seven and four individuals, respectively. Despite this, medication usage was a significant, albeit minor, contributor (*R*^2^ = 0.003) to microbial community composition, highlighting how xenobiotics can and do influence the gut microbiome (Jackson et al., 2018; Maier et al., 2018).

### MI bacterial profiles are comparable to those from Israeli donors

**Figure 2.**
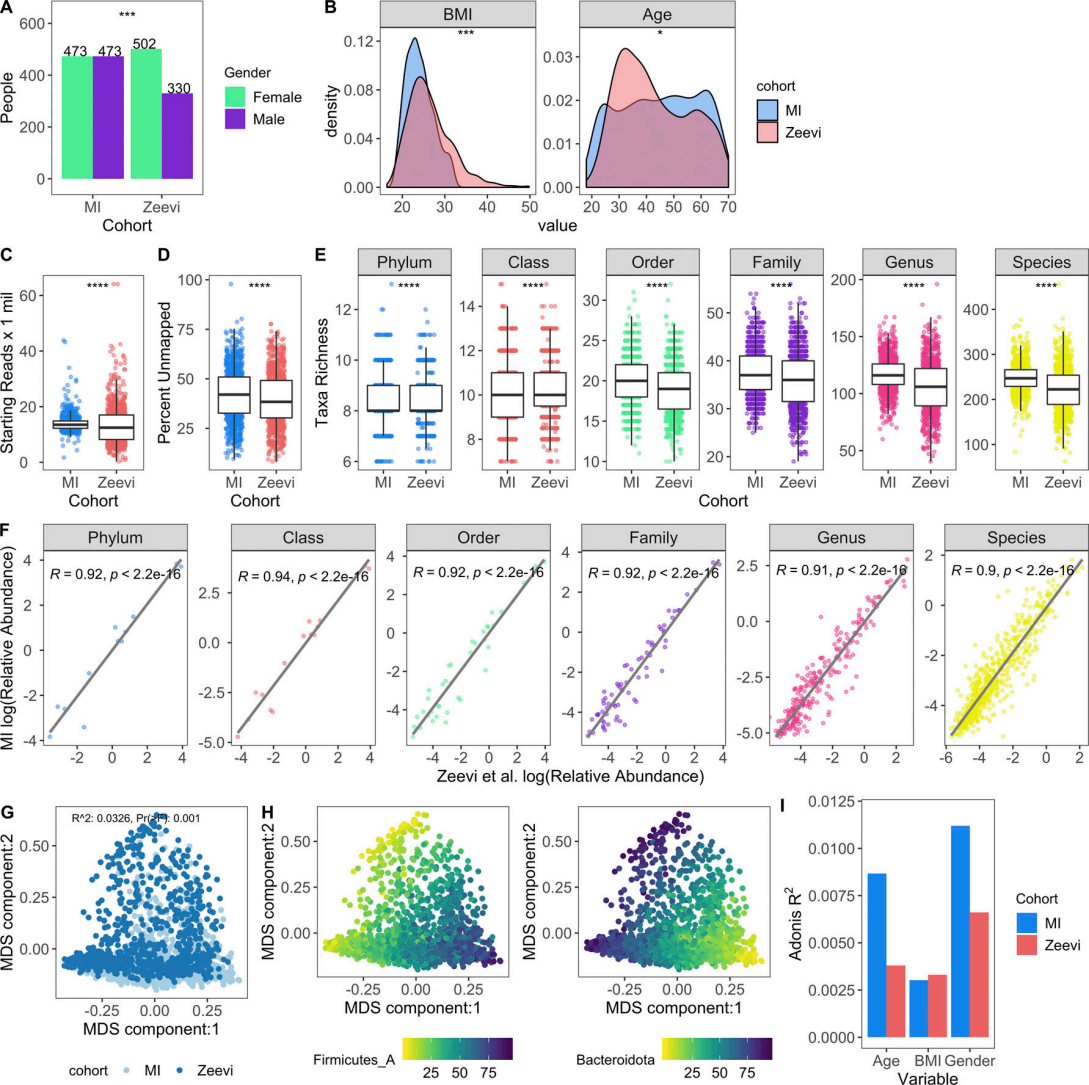
**Microbial profiles of MI donors in comparison to those from ****Zeevi et al. (2015)****.** **(A)** Bars indicate the number of females and males in both cohorts; P value by Fisher’s exact test; ***, P < 0.001. **(B)** Density plots show distribution of BMI and age in both cohorts; P value by Wilcoxon rank sum; *, P < 0.05; ***, P < 0.001. **(C)** Bar plots compare sequencing depth across cohorts. **(D)** Bar plots show percentage of reads unmapped after the Kraken2-GTDB pipeline. **(E)** Boxplots show richness across taxonomic levels. Each dot corresponds to one donor. **(C–E)** P values by Wilcoxon rank sum; ****, P < 0.0001. **(F)** Association of relative abundances of each taxon across taxonomic levels with Spearman correlation. Each dot corresponds to one taxon. Only taxa present in >5% of either cohort were considered. Log relative abundances are shown. **(G)** MDS ordination plot of all samples in both the MI and Zeevi cohorts. Each dot corresponds to one donor and is colored by the cohort. *R*^2^ indicates the amount of interindividual variation (calculated with BC) explained by the cohort and was calculated with the PERMANOVA test adonis. **(H)** MDS ordination plot of samples in both the MI and Zeevi cohorts. Each dot corresponds to one donor and is colored by the relative abundance of the phyla. **(I)** Bars indicate the amount of interindividual variation (calculated with BC) explained by each of the variables in each of the cohorts. See also Data S1, table 8.

To determine whether MI bacterial profiles were unique to this population or comparable with other non-European healthy cohorts, we ran our Kraken2/GTDB pipeline on 1,159 samples from 851 Israeli donors originally published by Zeevi et al. (2015) (Data S1, table 8), for whom age, sex, and body mass index (BMI) were provided (Fig. 2, A and B). After accounting for sequencing depth (mean read count: MI, 13.9 ± 2.9 million; Zeevi, 13.5 ± 7 million), we found that richness across taxonomic levels was consistently elevated in the MI samples, even though the percentage of unmapped reads was comparable (MI 42.2% versus Zeevi 39.6%; Fig. 2, C–E). More specifically, we identified on average 24 more species in samples from the MI donors than from the Zeevi cohort. In addition to potential technical and lifestyle reasons, this discrepancy could reflect the stricter inclusion and exclusion criteria, and thus the greater overall health, of the MI donors (Thomas et al., 2015; Zeevi et al., 2015).

On the whole, community composition, including relative taxa abundances and β diversity, was consistent across both cohorts (Fig. 2, F–H). Notably however, the contributions of age and sex to community composition were almost two times greater in MI than Zeevi (age: *R*^2^ = 0.0087 versus 0.0038; sex: *R*^2^ = 0.011 versus 0.0066; Fig. 2 I), highlighting how stratification of age and sex in the MI cohort provided enhanced statistical power to identify new correlations (Fig. 2, A and B; Zeevi et al., 2015). Despite technical differences, as well as geographic and cultural distinctions between these cohorts, our findings demonstrate a comparable makeup of the gut microbiome. This allowed us to use the Zeevi samples as a replication cohort to demonstrate the reproducibility of our findings in MI.

### *Prevotella* species are more abundant in male donors

**Figure 3.**
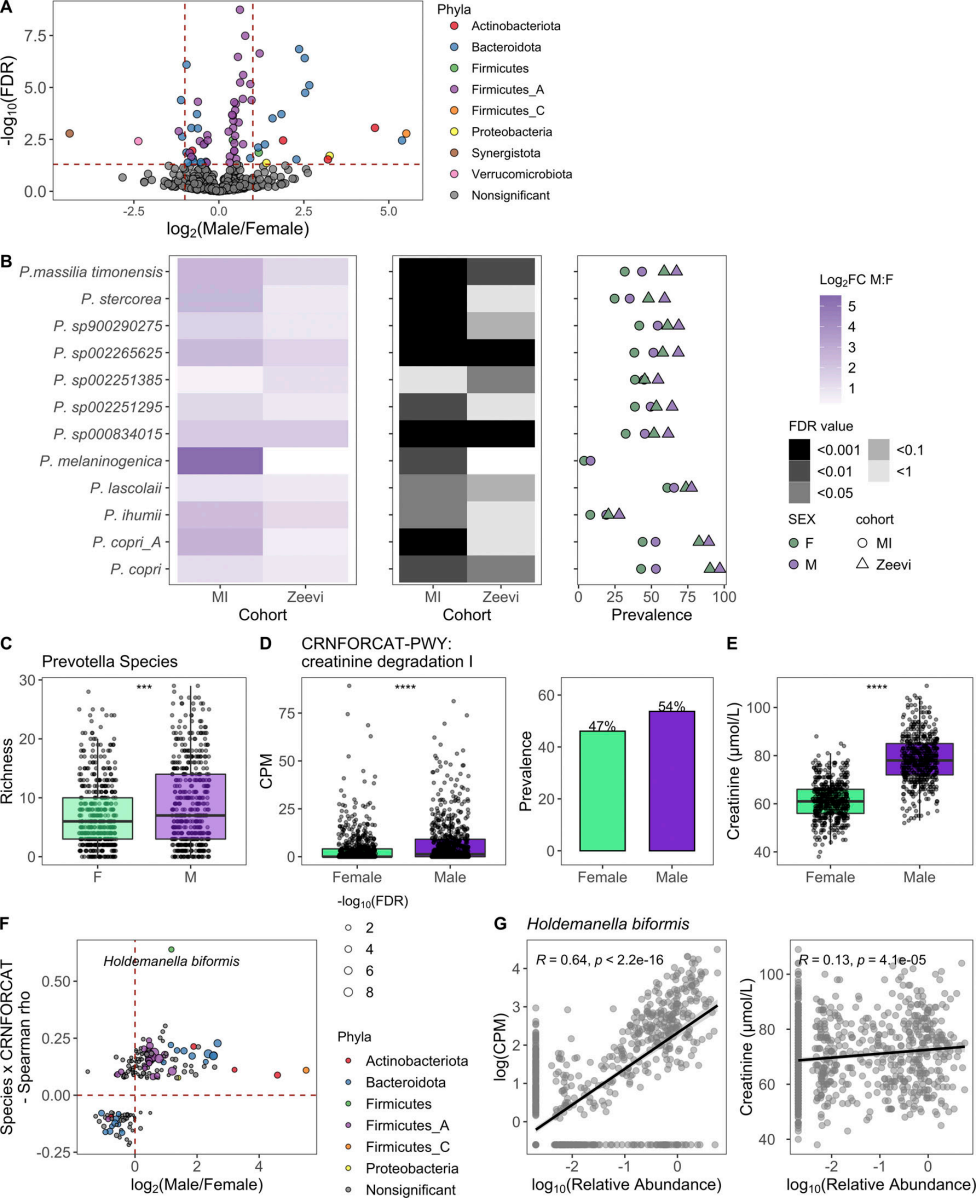
**Taxa, particularly *Prevotella*, were differentially present between males and females.** **(A)** Volcano plot of 485 abundant species, of which 71 were differentially abundant between males and females based on DESeq2 (FDR < 0.05). Each species is colored by its taxonomic phyla. **(B)** 12 *Prevotella* species were more abundant in males consistently across cohorts. Left: Log_2_ fold change (log_2_FC) of species in males versus females. Middle panel indicates the FDR value. Notably, even when a species was significant in only one cohort, the direction was consistent in the other. Right: Prevalence of each species. Color indicates the sex, while shape indicates the cohort. **(C)** Boxplots show richness of *Prevotella* species in males and females. **(D)** Abundance measure in counts per million (CPM) and prevalence of the pathway CRNFORCAT-PWY: creatinine degradation I in males and females. **(E)** Blood creatinine levels in males and females. **(C–E)** P values by Wilcoxon rank sum; ***, P < 0.001, ****, P < 0.0001. **(F)** In the scatter plot, dots represent the 189 species that were significantly correlated with CRNFORCAT-PWY. Of those, species that were significantly differentially abundant between males and females are colored by their phyla label. Species not associated with sex are colored gray. Size of the point corresponds to the −log_10_(FDR) of the species by sex correlation. **(G)** Scatter plots show the correlation of *Holdemanella biformis* relative abundance and CPM of CRNFORCAT (left) or circulating creatinine levels (right). Trend lines show 95% confidence intervals and were modeled with lm. Statistics based on Spearman correlation. See also Data S1, tables 9–11.

Given that sex and age were the variables most strongly associated with bacterial community composition in healthy individuals, we leveraged the statistical power of the MI cohort to explore which taxa were differentially abundant between sexes and across decades of life. To identify bacteria differentially abundant between the 473 females and 473 males, we conducted DESeq2 analysis using age and BMI as covariates (Love et al., 2014) on 485 abundant species (prevalence >5% and mean relative abundance >0.01%; Data S1, table 9). Of the 71 differentially abundant species (FDR < 0.05), 5 were more abundant in females, while 18 were more abundant in males, with log_2_ fold change >1 (Fig. 3 A). In total, 11 of 32 prevalent *Prevotella* species were more abundant in males than females, corresponding to a greater overall richness or number of unique *Prevotella* species in males (Fig. 3, B and C). Similarly, in the Zeevi cohort, five species of *Prevotella* were more abundant in males (Fig. 3 B and Data S1, table 10; Zeevi et al., 2015). Notably, even when a species was significantly differentially abundant between sexes in only one cohort, the direction of this trend was also consistent in the other, indicating that higher *Prevotella* abundance in males compared with females is a biological phenomenon consistent across multiple species and populations. This information increases the granularity of trends presented in two previous studies, one that identified *Bacteroides*-*Prevotella* as broadly more abundant in males than females based on 16S rRNA-targeted oligonucleotide probes (Mueller et al., 2006), and another that found males were three times more likely to have an enterotype consisting of fewer *Bacteroides* and higher *Prevotella* (Ding and Schloss, 2014). Although the factors driving preferential colonization of *Prevotella* in males are unknown, from these data we could generate hypotheses surrounding the roles of gonadal hormones and microbial community composition. In support of this hypothesis, in a longitudinal study of the oral microbiome, serum levels of testosterone in boys and estradiol and progesterone in girls were positively correlated with levels of *Prevotella intermedia* (Nakagawa et al., 1994).

When considering 364 prevalent metabolic pathways (prevalence >5%), we identified 65 (FDR < 0.05) that were differentially abundant between the sexes (Data S1, table 11). Of those, the pathway CRNFORCAT-PWY: creatinine degradation I was the most strongly enriched in men (Fig. 3 D). Biologically, this is consistent with men having higher blood levels of creatinine (Fig. 3 E). Across both sexes, but not in each individually, circulating creatinine levels were significantly associated with the abundance of the CRNFORCAT-PWY pathway (both sexes: Spearman ρ = 0.087, P = 0.0014; men: ρ = 0.028, P = 0.47; women: ρ = −0.047, P = 0.22). Of species positively associated with this pathway, many were more abundant in males, including the top species *Holdemanella biformis*, which was also correlated with circulating creatinine levels (Spearman ρ = 0.13, P = 4.1 × 10^−5^; Fig. 3, F and G; and Data S1, table 11). Overall, this exemplifies how adaptation to use available nutrients may influence microbiome composition.

### The gut microbiome is dynamic across decades of life

It is well characterized that the composition of the gut microbiome differs dramatically between newborns and adults, with the neonatal microbiome transitioning to a more adult-like state upon consumption of solid food and cessation of breastfeeding (Bäckhed et al., 2015; Stewart et al., 2018). However, how the gut microbiome changes throughout adult life has been primarily studied in smaller cohorts by culturing or 16S rRNA analyses (An et al., 2018). The design of the MI cohort provides a unique opportunity to explore how in the absence of underlying disease the gut microbiome is dynamic across the adult decades (20–69 yr old).

**Figure 4.**
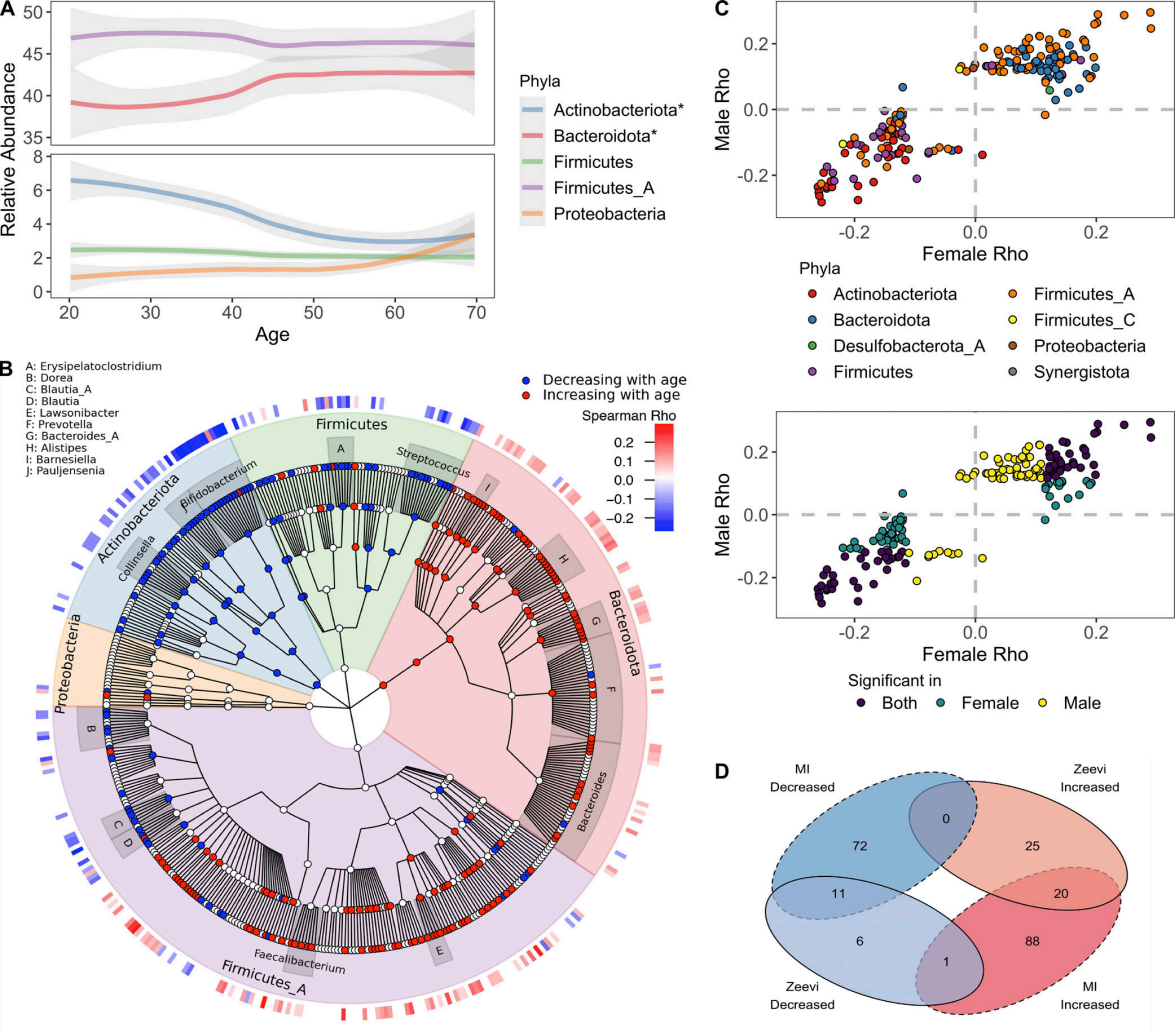
**Bacterial profiles are dynamic across decades of life.** **(A)** Abundance of the five most abundant phyla across decades of life. Curves show 95% confidence intervals and were modeled with LOESS regression. Stars in the figure legend indicate those phyla statistically associated with age. **(B)** GraPhlAn taxonomic tree of the 454 species in the top five phyla found at significant prevalence and abundance across all donors. Relative abundance of taxa in red were decreased with age, while those in blue increased. Association between taxa relative abundance and age was determined by Spearman correlation (FDR < 0.05). The heatmap on the outer ring indicates the strength of the correlation (Spearman ρ). Genera with at least four species associated with age are labeled. **(C)** Scatter plots compare the Spearman ρ values of bacteria ~ age for males and females, one point = one species. In the bottom plot, points are colored based on whether the correlation was significant (FDR < 0.05) in males, females, or both. In the top plot, points are colored based on the phyla designation of the species. **(D)** Venn diagram comparing the bacterial species statistically (FDR < 0.05) associated with age in the MI and Zeevi et al. (2015) cohorts. See also Data S1, tables 12 and 13.

**Figure 5.**
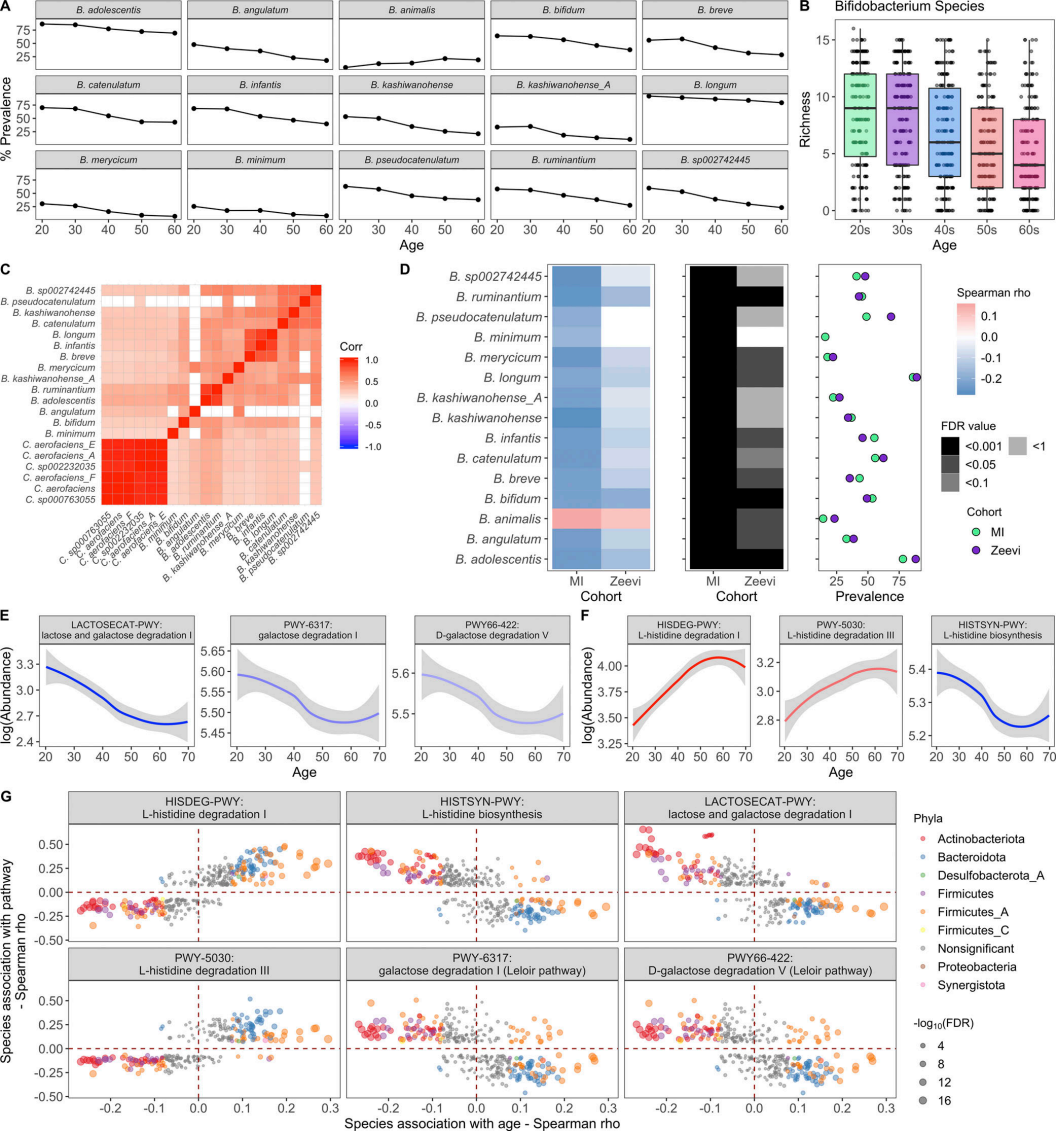
***Bifidobacterium* species and *Bifidobacterium*-associated pathways decrease with age.** **(A)** Lines indicate the percent prevalence of 15 *Bifidobacterium* species across the different decades. Only *Bifidobacterium* species significantly associated with age are shown. **(B)** Boxplots show richness of *Bifidobacterium* species in donors grouped by their age. **(C)** Correlation plot of *Bifidobacteria* species (prevalence >30%) and their cocorrelated *Collinsella* species (Spearman ρ > 0.4). **(D)** 15 *Bifidobacterium* species were associated with age across cohorts. Left: Correlation of species with age (Spearman ρ). Middle: FDR value. Notably, even when a species was significant in only one cohort, the trend was consistent in the other. Right: Prevalence of each species. Color indicates the cohort. **(E)** Abundance (counts per million [CPM]) of lactose/galactose degradation pathways decreased with age. Curves show 95% confidence intervals and were modeled with LOESS regression. **(F)** Abundance (CPM) of l-histidine degradation/synthesis pathways associated with age. Curves show 95% confidence intervals and were modeled with LOESS regression. **(G)** For the pathways correlated with age in E and F, in the scatter plot, dots represent the species that were significantly correlated with that pathway. Of those, species that were also significantly correlated with age are colored by their phyla label. Species not associated with age are colored gray. Size of the point corresponds to the −log_10_(FDR) of the species by age correlation. See also Data S1, tables 12–14.

In total, we found that 40% of abundant species (192/485) were differently abundant by donor age (Spearman correlations of age by taxa relative abundance, FDR < 0.05); consistent results were identified with linear models taking into account sex and BMI (Data S1, table 12). Notably, two of the top five phyla (Bacteroidota and Actinobacteriota) experienced shifts in relative abundance across the decades (Fig. 4, A and B). Transitions were most pronounced around 40–50 yr old, a time span when many people experience the preclinical stages of chronic diseases, and women begin to experience hormonal changes associated with onset of menopause; average age in this cohort 50 ± 4.2 yr. Across phyla, the correlations with age were conserved across sexes and cohorts (Fig. 4, C and D). For example, relative abundances of Bacteroidota species were primarily increased with age, while Actinobacteriota, including 15 species of *Bifidobacterium*, were decreased with age (Fig. 4 B). This gradual decline of *Bifidobacterium* was true in terms of both relative abundance as well as presence/absence of individual species (Fig. 5 A). In fact, the overall richness or number of unique *Bifidobacterium* species present in an individual steadily declines throughout life (Fig. 5 B). Notably, many *Collinsella* species that were positively correlated with *Bifidobacterium* were also decreased with age (Figs. 4 B and 5 C).

The decline in *Bifidobacterium* prominence with age is particularly interesting in light of *Bifidobacterium* being the dominant bacteria in many newborns, gradually decreasing as infants cease breastfeeding (Stewart et al., 2018). The association of *Bifidobacterium* and old age indicates that the loss of *Bifidobacterium* occurs not only in infants, but continues throughout adulthood (An et al., 2018; Biagi et al., 2010, 2016; Kato et al., 2017; Mueller et al., 2006). Similar to our findings associating *Prevotella* and sex (Fig. 3 B), we built on previous lower-resolution findings to reveal that the trend was consistent across numerous species within the genera and across cohorts (Fig. 5 D and Data S1, table 13), highlighting how the phenomenon is intrinsic to this species. Notably, the only exception, *Bifidobacterium animalis*, is a common probiotic-associated strain (Turroni et al., 2009), rather than a persistent colonizer. Moving forward, comparative genomic analyses between these different species could reveal features associated with colonization in older adults.

We then focused our attention on 364 prevalent microbial pathways (prevalence >5%) and identified 108 that correlated with age (Data S1, table 14), of which 31 were increased and 77 were decreased (FDR < 0.05), including several lactose and galactose degradation pathways (Fig. 5 E). Consistent with the previous results, these pathways were strongly correlated with species in the *Bifidobacterium*, *Collinsella*, and *Blautia* genera (Fig. 5 G and Data S1, table 14). Lower levels of lactose/galactose degradation may explain increased lactose intolerance in older adults and presents a possible opportunity for microbial therapeutic intervention (Gingold-Belfer et al., 2020; Savaiano et al., 2013). Notably in this cohort, the abundance of these pathways was not associated with consumption of dairy products, e.g., milk, cheese, and yogurt (Spearman ρ > 0.3).

Other pathways associated with age were related to l-histidine. In this case, pathways for l-histidine biosynthesis were decreased with age, while those for degradation were increased (Fig. 5 F). Concordantly, the biosynthesis pathway was positively correlated with species decreased with age, while degradation pathways were correlated with species increased with age (Fig. 5 G and Data S1, table 14). In total, these results indicate that gut l-histidine levels may be decreased in older adults, which could lead to an altered immune state, as l-histidine metabolites have been demonstrated to influence colonic inflammation (Gao et al., 2017). Overall, understanding the multitude of microbial correlations with age is incredibly important for appreciating the microbial shifts observed in diseases affecting older individuals.

### Short-term stability is variable across donors

**Figure 6.**
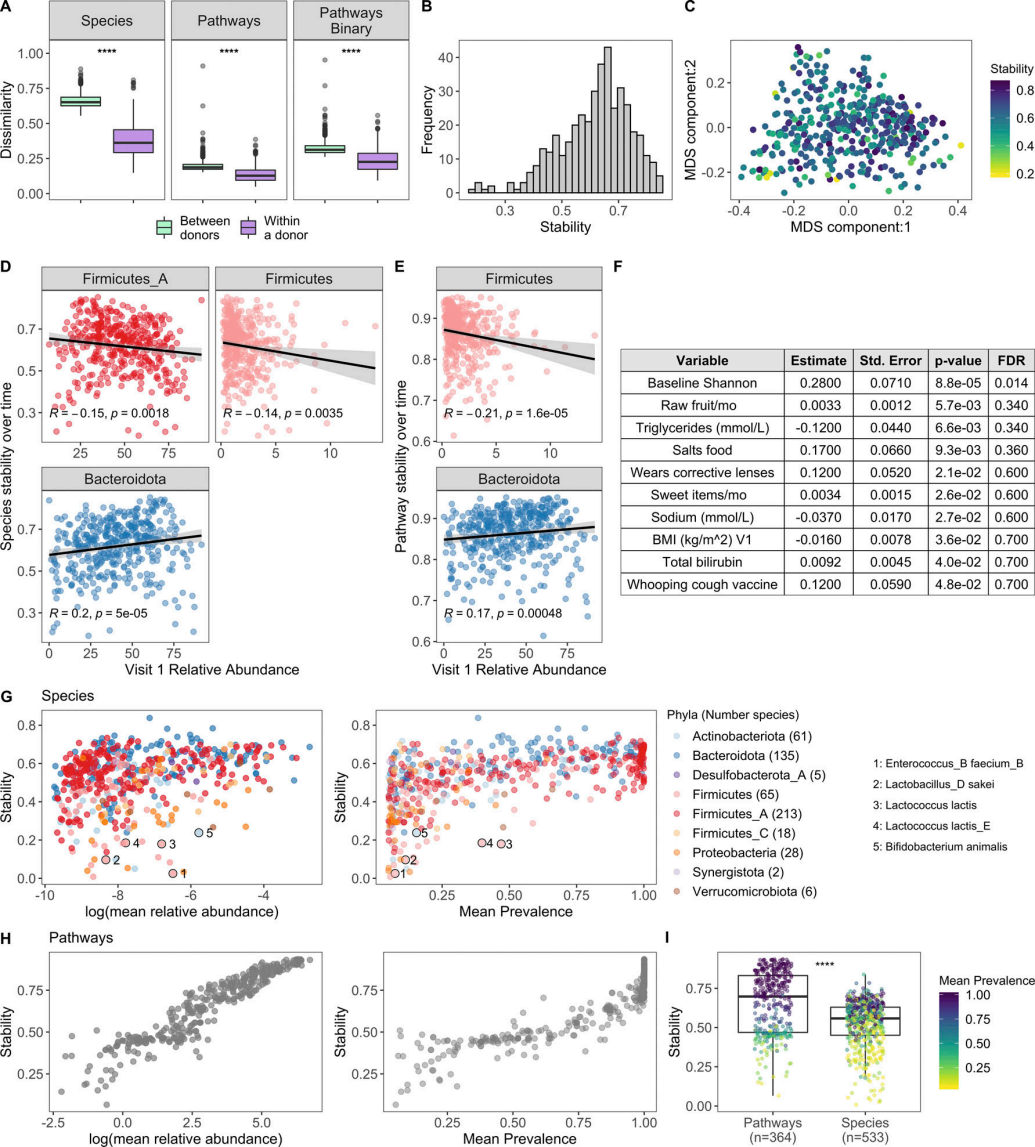
**Bacterial profiles are stable over the short term; however, the degree of stability is variable across donors.** **(A)** Boxplots of species BC distances (left), pathway BC distances (middle), and pathway binary Jaccard distances (right) between donors (*n* = 946, for each individual, the average distance from all other individuals at V1 was used) and within a donor over time (*n* = 413, time between samples = 17 ± 3.3 d); 1 = samples are completely different, 0 = samples are identical. ****, P < 2.2 × 10^−16^ by Wilcoxon rank sum. **(B)** Histogram of 413 donors’ species stability (1 − BC). **(C)** MDS plot of BC distance of bacterial species composition. Each dot corresponds to one donor who had a second sample. Color indicates longitudinal species stability of that donor (1 − within-sample BC). **(D)** Scatter plots show the top phyla associated with species stability (FDR < 0.05). **(E)** Scatter plots show the top phyla associated with pathway stability (FDR < 0.05). **(D and E)** Each point corresponds to one donor with a longitudinal sample (*n* = 413). Trend lines show 95% confidence intervals and were modeled with lm. Statistics based on Spearman correlation. **(F)** Results of the series of GLM fits aimed at identifying factors associated with intraindividual species stability. Only factors with P value < 0.05 are shown. Std., standard. **(G)** Scatter plots show the stability of individual species (1 − BC) by their mean baseline relative abundance and prevalence. Each point corresponds to a bacterial species and is colored by the species Phyla. 533 species with mean prevalence >5% are shown. **(H)** Scatter plots show the stability of individual pathways (1 − BC) by their mean baseline relative abundance and prevalence. Each point corresponds to a pathway. 364 pathways with mean prevalence >5% are shown. **(I)** Boxplots compare stability of individual species and pathways (1 − BC). Points are colored by the mean prevalence of the species or pathway in both samples. ****, P < 2.2 × 10^−16^ by Wilcoxon rank sum. See also Data S1, tables 15–18.

To complement our cross-sectional study of the microbiome across the decades, we leveraged longitudinal sampling of roughly half the cohort (*n* = 413) to study short-term (17 ± 3.3 d) dynamics within an individual in the absence of antibiotic exposure. By comparing species BC distances within and between individuals, we found that in the short term, intraindividual differences were less than the interindividual ones (Fig. 6 A). This is consistent with previously published findings (Costello et al., 2009; Flores et al., 2014; Mehta et al., 2018) and also reflected the analysis of relative abundance and presence/absence of metabolic pathways (Fig. 6 A). While within an individual species and pathway, stabilities were highly correlated (Spearman ρ = 0.75, P < 2.2 × 10^−16^), differences between donors were less dramatic at the pathway level, reflecting the more conserved nature of annotated metabolic pathways versus species profiles across individuals (Human Microbiome Project Consortium, 2012).

Although stability was the norm, the degree of species stability (quantified as 1 − BC distance; Mehta et al., 2018) was variable across the 413 donors (Fig. 6, B and C), and as such we investigated which microbial and metadata features underlie this personalized stability trait. Using Spearman correlations, we identified relative abundances of the phyla Firmicutes and Firmicutes_A as enriched at baseline in the less stable donors, while Bacteroidota was higher in donors with greater species stability over time (Fig. 6 D and Data S1, table 15; Flores et al., 2014). Notably, similar trends also were observed when analyzing pathway stability (Fig. 6 E and Data S1, table 15). This is consistent with observations that spore-forming bacteria, including many Firmicutes species, are intrinsically less stable (Kearney et al., 2018). Using the generalized linear model (GLM), we compared for the first time how stability is influenced by an extensive list of metadata variables. This revealed that BMI and circulating triglyceride levels were negatively associated with stability, while conversely, consumption of sweet items (e.g., chocolate, sweets, honey, and jam) and raw fruit were positively associated (Fig. 6 F and Data S1, table 16), concordant with diet being a key determinant of human gut microbiome variation (Johnson et al., 2019). While the previous results were only marginally significant (P < 0.05), consistent with previous findings (Flores et al., 2014; Mehta et al., 2018) and ecological theory (McCann, 2000; Schindler et al., 2010; Tilman, 1999), baseline species Shannon diversity was positively associated with stability (FDR = 0.014; Fig. 6 F), i.e., individuals with more diverse communities were more resilient to change than individuals with lower diversity.

We then applied the BC metric to calculate stability (1 – BC) of individual species and pathways (Data S1, tables 17 and 18; Faith et al., 2013; Franzosa et al., 2015). For both species and pathways, we found that stability was strongly associated with mean abundance and prevalence across donors (Fig. 6, G and H). For example, the Firmicutes *Enterococcus_B faecium_B* had a mean abundance of 0.15%, prevalence 7.5%, and low stability (0.025). Additionally, many species known to be present in yogurt and probiotics were also highly unstable, e.g., *Lactobacillus_D sakei*, *Lactococcus lactis*, and *B*.* animalis* (Fig. 6 G; Fijan, 2014), in agreement with previous observations that probiotics often face colonization resistance (Zmora et al., 2018). Comparison of overall stability of pathways and species revealed that individual pathways were on average more stable than individual species (Fig. 6 I), consistent with pathways being more conserved across individuals (Fig. 6 A). Moving forward, these data can be leveraged to prioritize microbial pathways/species that will make reliable biomarkers as well as persistent colonizers if incorporated into a microbial therapeutic.

### Across cohorts, patients with non-GI cancers have altered gut microbial communities

**Figure S4.**
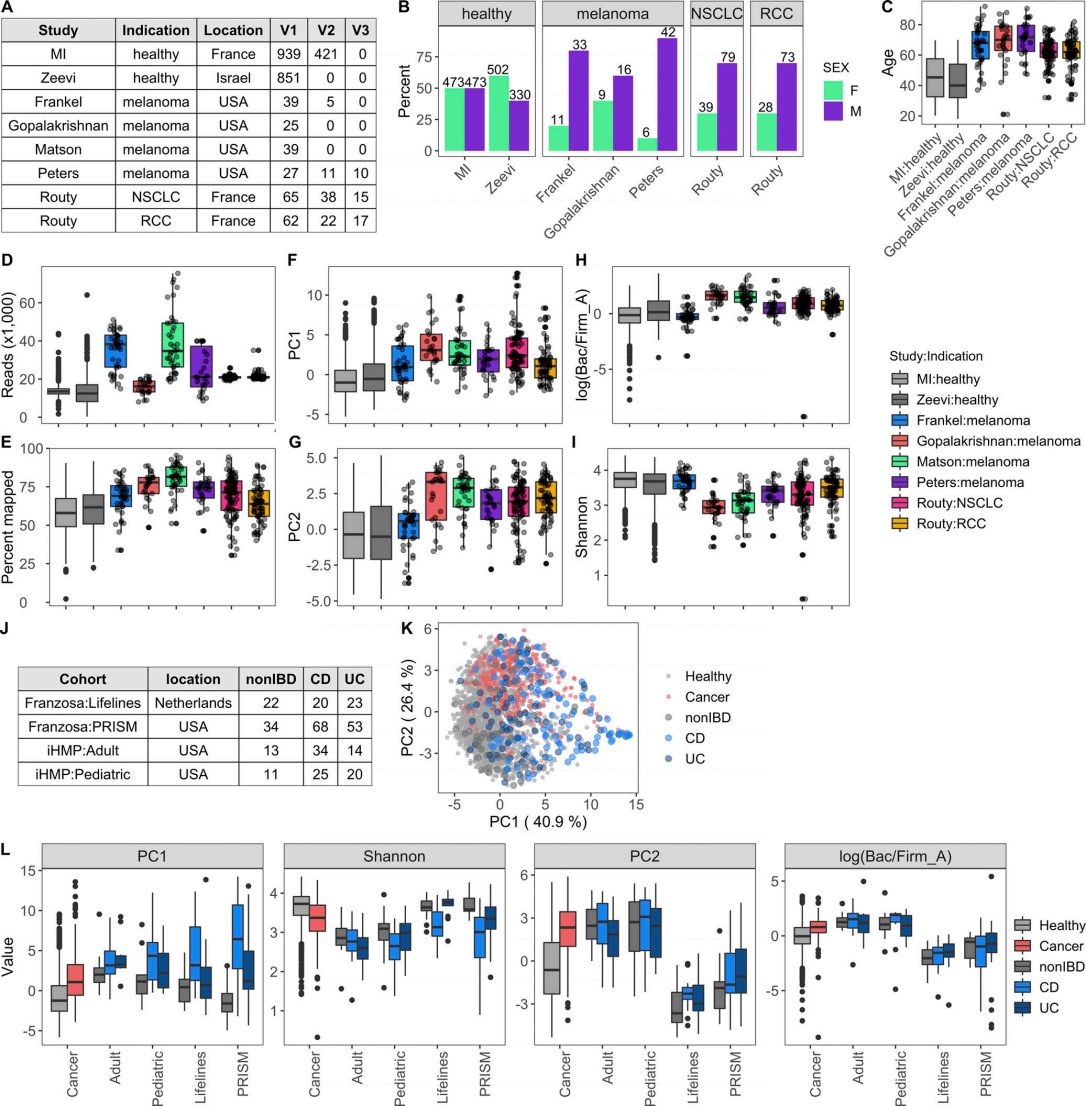
**Across studies and indications, cancer patients have altered gut bacterial profiles.** Related to Fig. 7. **(A)** Table indicating the studies included in all cancer versus healthy analyses. For all but the longitudinal analysis, one sample per patient was included. When available, the baseline sample was prioritized. For cancer patients, V1 = baseline, pretreatment; V2-Frankel = within 1 mo of starting ICT; V2-Peters = week 6; V2-Routy = after second injection, ∼1 mo; V2-Peters = week 12; V3-Routy = after fourth injection, ∼2 mo. **(B)** Bars indicate percentage of males and females in each of the studies. Number indicates the actual number of males and females. Sex data were not provided by Matson et al. (2018). **(C)** Boxplots compare age of donors across the studies. Age data were not provided by Matson et al. (2018). **(D–I)** Boxplots compare values indicated on the y axis across the healthy and cancer cohorts. Color corresponds to the study. **(J)** Table indicating the number of donors in each of the IBD cohorts from the two studies. Patients in iHMP <20 yr old were grouped in the pediatric cohort. **(K)** PC plot of BC distance of bacterial species composition of donors in Fig. 7 A as well as donors in the IBD cohorts. For the HMP samples, multiple samples from the same donor were aggregated together, so there is only one dot per donor. Color corresponds to the health status. **(L)** Boxplots compare PC1, PC2, Shannon, and Bacteroidota (Bac)/Firmicutes_A (Firm) ratios of samples in K. On the x axis, “cancer” is all the samples in Fig. 7; the remaining are the IBD cohorts in J. CD, Crohn’s disease; ICT, immune checkpoint inhibitor; NSCLC, non–small cell lung cancer; RCC, renal cell carcinoma; UC, ulcerative colitis.

**Figure 7.**
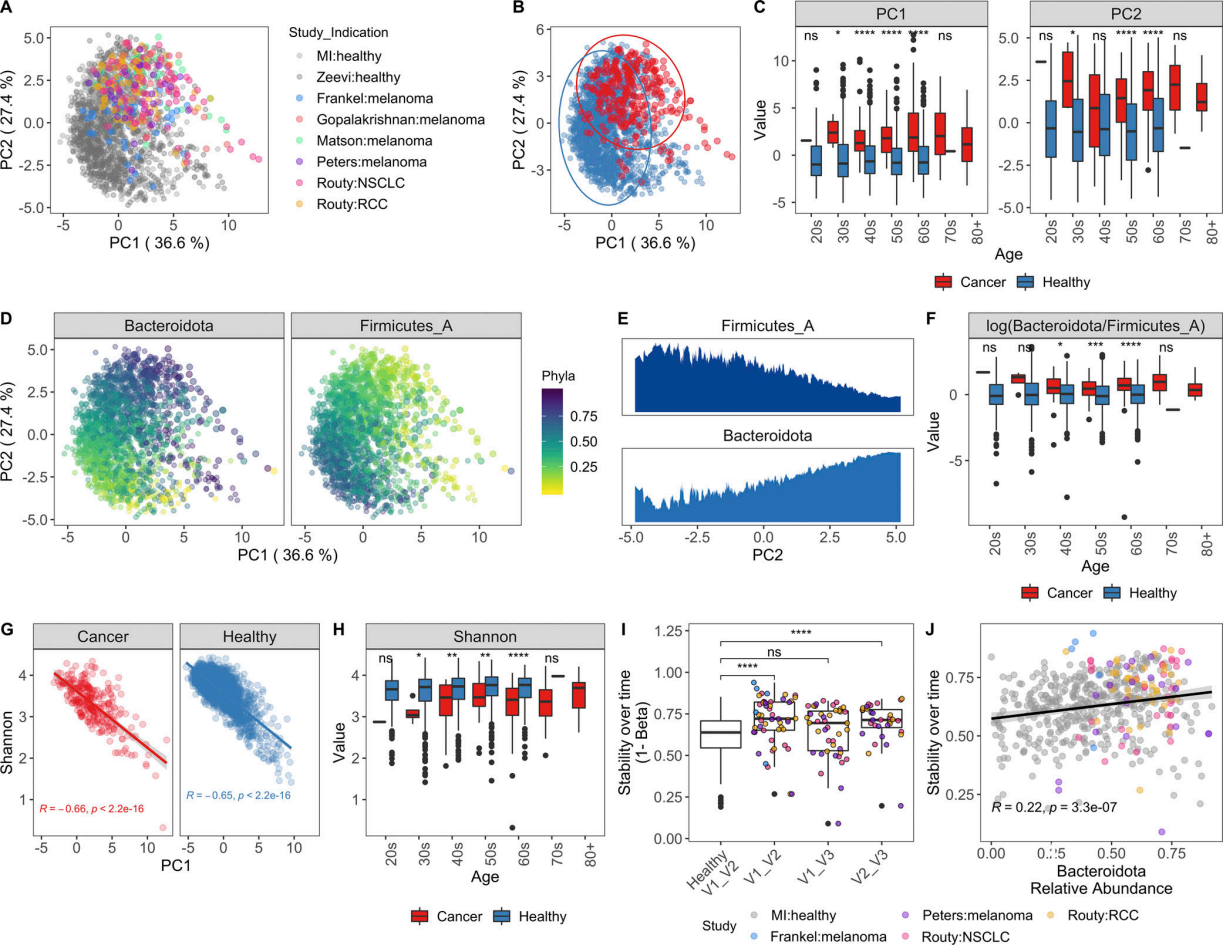
**Cancer patients have altered gut bacterial profiles associated with higher Bacteroidota/Firmicutes_A ratios and lower Shannon diversity.** **(A)** PC plot of BC distance of bacterial species composition of one sample per donor in the table in Fig. S4 A. When available, the baseline sample was prioritized. Each dot corresponds to one donor, while color indicates the study and indication. **(B)** Same PC analysis plot as A, with dots colored by health status of the donor. Red, cancer; blue, healthy. **(C)** Boxplots compare PC1 and PC2 values of samples from cancer patients and healthy donors stratified by age. **(D)** Same PC analysis plot as A with dots colored by relative phyla abundance. **(E)** Density plots of Bacteroidota and Firmicutes_A were generated using a moving average of the abundance of the phyla within the communities along PC2, with a scale from 0 to the maximum moving average. **(F)** Boxplots compare the log(Bacteroidota/Firmicutes_A) ratio of samples from cancer patients and healthy donors stratified by age. **(G)** Scatter plots show PC1 versus Shannon for cancer patients and healthy donors. Trend lines show 95% confidence intervals and were modeled with lm. Statistics based on Spearman correlation. **(H)** Boxplots compare bacterial Shannon diversity of samples from cancer patients and healthy donors stratified by age. **(I)** Boxplots of species stability (1 − BC distance) within a donor over time points indicated in the x axis. For cancer patients, V1 = baseline, pretreatment; V2-Frankel = within 1 mo of starting ICT; V2-Peters = week 6; V2-Routy = after second injection, ∼1 mo; V2-Peters = week 12; V3-Routy = after fourth injection, ∼2 mo. **(J)** Scatter plots shows Bacteroidota abundance versus stability (1 − BC distance) for cancer and healthy patients. Trend lines show 95% confidence intervals and were modeled with lm. Statistics based on Spearman correlation. For C, F, H, and I, ns, not significant (P > 0.05); *, P < 0.05; **, P < 0.01; ***, P < 0.001; ****, P < 0.0001 by Wilcoxon rank sum with FDR correction. See also Fig. S4. ICT, immune checkpoint inhibitor; NSCLC, non–small cell lung cancer; RCC, renal cell carcinoma.

Given our success integrating results of the MI cohort with those from Zeevi et al. (2015), we sought to determine whether similar congruence was observed in the gut microbiome of cancer patients. We focused on those with non-GI tumors, for whom recent publications have demonstrated associations between microbiome composition and positive responses to checkpoint inhibitors (Frankel et al., 2017; Gopalakrishnan et al., 2018; Matson et al., 2018; Peters et al., 2019; Routy et al., 2018). In contrast to colorectal cancer (Thomas et al., 2019), there remains a large gap in our knowledge detailing how the microbiome composition of cancer patients with non-GI indications compares to that of healthy donors. To investigate this, we applied our Kraken2/GTDB pipeline to an additional 375 samples from 283 cancer patients across five published cohorts (Frankel et al., 2017; Gopalakrishnan et al., 2018; Matson et al., 2018; Peters et al., 2019; Routy et al., 2018; Fig. S4, A–E). Despite technical and geographic differences, we identified that cancer patients have significantly altered gut bacterial communities compared with their healthy counterparts, as quantified by differences in principal coordinates (PCs) 1 and 2 (Fig. 7, A and B). When comparing datasets across cohorts, even those processed with identical analytical methods, there is always a risk of differences being driven by technical artifacts, for example collection or sequencing method. However, in this case, the differences between healthy donors and those with cancer were consistent across age groups (Fig. 7 C) and diverse cohorts (Fig. S4, F and G), supporting our conclusion.

Specifically, when compared with healthy age-matched controls, cancer patients had increased Bacteroidota/Firmicutes_A ratios (driver of PC2; Fig. 7, D–F) and decreased Shannon diversity (driver of PC1; Fig. 7, G and H). Across measures (Fig. S4, F–I), patients in the Frankel et al. (2017) cohort were closer to healthy donors than patients in the other cancer cohorts. Given that the indication, geographic location, and age distribution were similar to Gopalakrishnan et al. (2018), additional information is needed to understand why this may be the case. To determine if these microbial shifts were consistent in individuals with a chronic disease other than cancer, we analyzed an additional 520 samples from 257 patients with IBD, either ulcerative colitis or Crohn’s disease, and 80 controls from two published studies (Fig. S4 J; Franzosa et al., 2019; Schirmer et al., 2018). While PC1 values were increased and Shannon values were consistently reduced in the donors with IBD, PC2 varied more by study than health status, with the integrative Human Microbiome Project (iHMP) samples consistently having higher PC2 and Bacteroidota/Firmicutes_A ratios than donors from the Lifelines and Prism cohorts (Fig. S4, K and L). This indicates that while high Bacteroidota/Firmicutes_A may be a conserved feature of the gut microbiome in patients with cancer, this trend is not found in other chronic diseases, as suggested by the results from two IBD cohorts.

To determine if receiving checkpoint inhibitors dramatically alters the gut microbiome, we compared longitudinal stability of the 60 patients with an on-treatment sample to that of the MI donors (Fig. S4 A). Counter to their low Shannon diversity values, we found that cancer patients on checkpoint inhibitor treatment were on average significantly more stable than the healthy MI donors (Fig. 7 I), consistent with our observation that Bacteroidota levels are associated with greater community stability (Fig. 7 J). Although these patients have relatively stable microbial communities on treatment, checkpoint inhibitors are rapidly being tested in combination with other agents (Tang et al., 2018), including chemotherapeutics, which themselves have been shown to alter microbial communities (Montassier et al., 2015). Therefore, additional studies are needed to understand if and how these emerging therapeutic combinations alter the gut microbiome.

**Figure S5.**
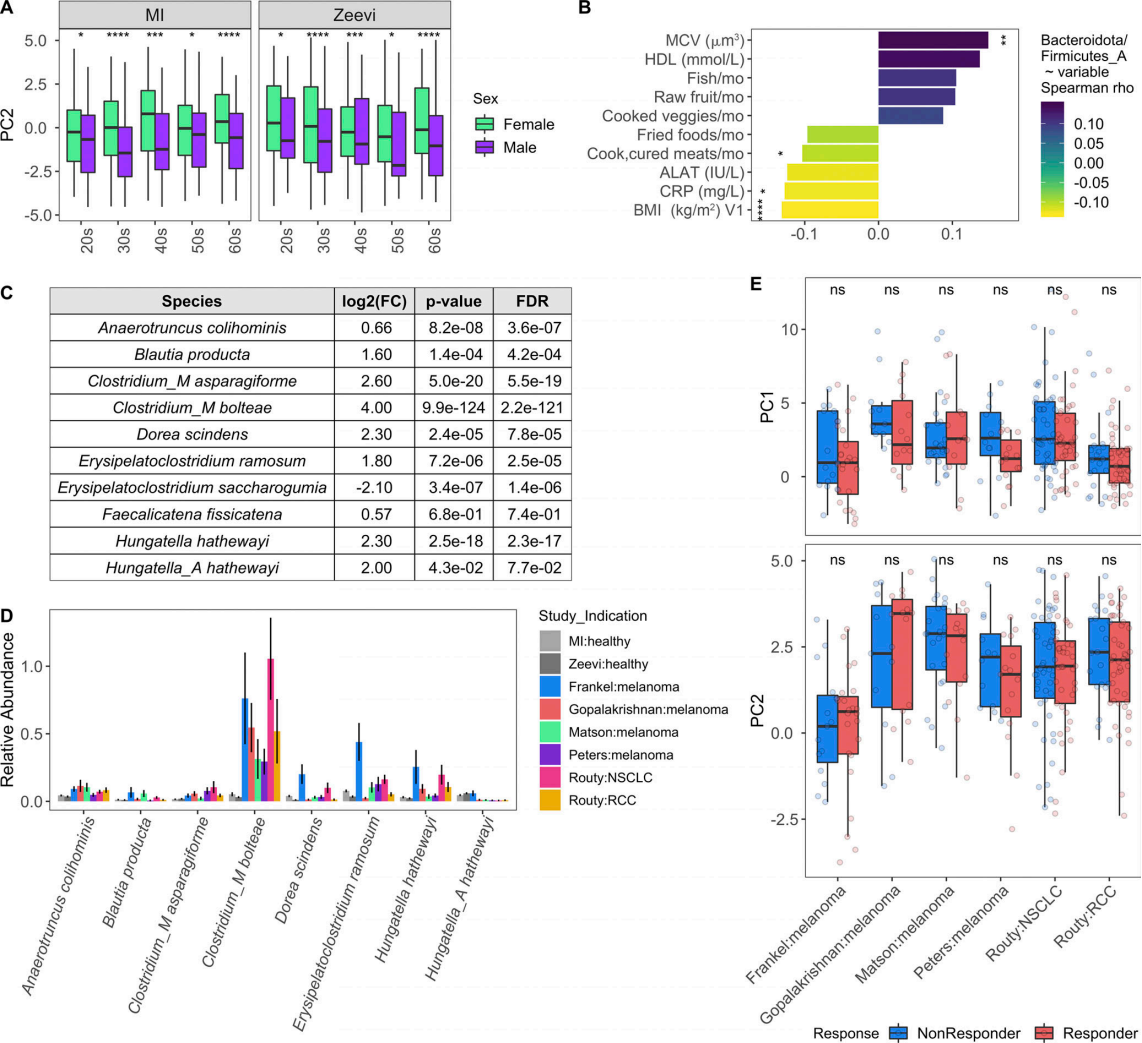
**Variables associated with cancer-like microbiome.** Related to Fig. 8. **(A)** Boxplots compare PC2 values of samples from males and females stratified by study and age. *, P < 0.05; ***, P < 0.001; ****, P < 0.0001 by Wilcoxon rank sum with FDR correction. **(B)** Length and color of the bars indicates the Spearman ρ value of each of the variables by ratio of Bacteroidota/Firmicutes_A. Only those variables with an FDR < 0.05 are shown. Stars indicate the P value after correcting for sex and age in a linear model. *, P < 0.05; **, P < 0.01; ****, P < 0.0001. **(C)** Table shows DESeq2 results for species identified by Atarashi et al. (2013) and Narushima et al. (2014) as potent inducers of regulatory T cells. Only species assigned a name in Narushima et al. (2014) that could then be linked to a representative in our database were included. **(D)** For the most abundant species in C, bars indicate the mean relative abundance of the species in each study. Error bars indicate mean ± SE. **(E)** Boxplots compare PC1 and PC2 values of samples from responders and nonresponders across studies. P values by Wilcoxon rank sum with FDR correction; ns, not significant (P > 0.05). Definitions of responders and nonresponders by study: Frankel R, response, stable disease; Frankel NR, progressed; Matson R, complete response, partial response; Matson NR, stable disease, progressed; Routy R, complete response, partial response, or stable disease; Routy NR, progressed or died; Gopalakrishnan R, complete response, partial response, or stable disease for ≥6 mo; Gopalakrishnan NR, progressed or stable disease <6 mo; Peters R, did not progress; Peters NR, progressed. See also Data S1, tables 19–21. NSCLC, non–small cell lung cancer; RCC, renal cell carcinoma.

**Figure 8.**
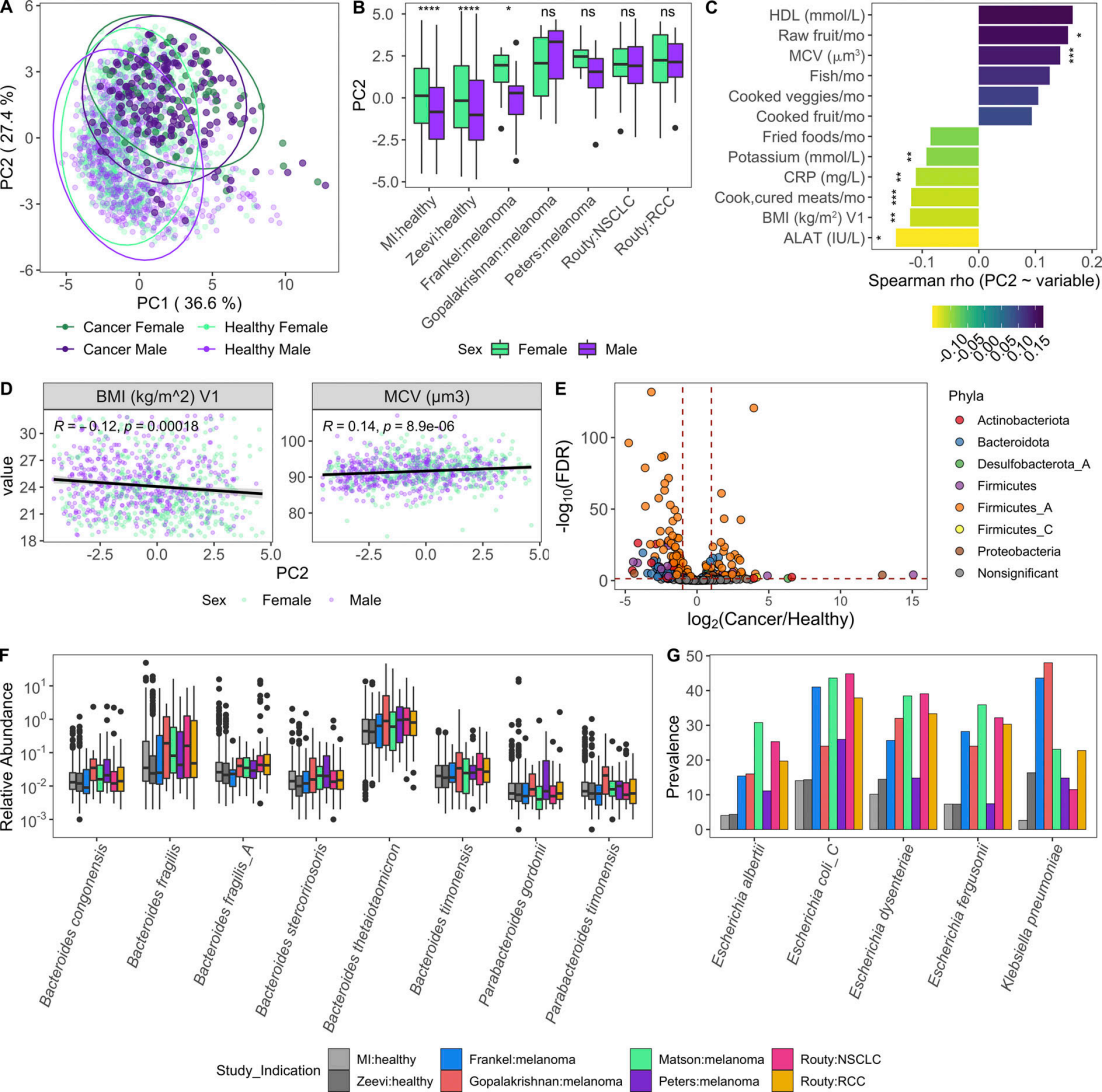
**Variables associated with a more cancer-like microbiome.** **(A)** Same PC analysis plot as Fig. 7 A. Each dot corresponds to one donor, while color indicates health status of the donor and their sex. **(B)** Boxplots compare PC2 values of samples from males and females stratified by study; ns, not significant (P > 0.05); *, P < 0.05; ****, P < 0.0001 by Wilcoxon rank sum with FDR correction. **(C)** Length and color of the bars indicates the Spearman ρ value of each of the variables by PC2. Only those variables with an FDR < 0.05 are shown. Stars indicate the P value after correcting for sex and age in a linear model. *, P < 0.05; **, P < 0.01; ***, P < 0.001. **(D)** Scatter plots show PC2 value versus variables from C. Trend lines show 95% confidence intervals and were modeled with lm. Statistics based on Spearman correlation. **(E)** Volcano plot of species differentially abundant between cancer patients and healthy donors based on DESeq2. To ensure that results were not driven by rare species, particularly in the large healthy cohorts, we first removed any species not present in at least three of the cancer cohorts at >10% prevalence, and then kept only the 448 species with a mean relative abundance >0.01%. Each species is colored by its taxonomic phyla. **(F)** Boxplot of Bacteroidota species more abundant in cancer patients than healthy donors. Y axis is on log10 scale. **(G)** Prevalence of selected Enterobacteriaceae species across healthy and cancer cohorts. Color corresponds to study. See also Fig. S5 and Data S1, tables 19–21. NSCLC, non–small cell lung cancer; RCC, renal cell carcinoma.

In the absence of extensive metadata for the patients with cancer, we leveraged the detailed characterization of the MI cohort to understand within healthy donors which factors contribute to a more “cancer-like” microbiome, as characterized by greater PC1 and PC2 values. Unlike PC1, primarily driven by Shannon diversity (Fig. 7 G), PC2 was associated with multiple factors (Data S1, table 19). Notably, PC2 was consistently elevated in females across the two healthy cohorts (Fig. S5 A). By contrast, these differences were diminished in the cancer cohorts (Fig. 8, A and B), where the average PC2 value was higher despite an enrichment of male patients (Fig. S4, B and G). From this, we can hypothesize that factors driving gut microbiome differences between males and females may be diminished in cancer patients.

After controlling for sex and age, we found that factors generally associated with good health, such as fruit consumption and mean corpuscular volume, were elevated with PC2, while factors associated with poor health, including BMI and circulating levels of the liver proteins alanine aminotransferase, and C-reactive protein, were lower in donors with a more cancer-like, PC2-high microbiome (Fig. 8, C and D; and Data S1, table 19). Many of these correlations were also true when looking at the Bacteroidota/Firmicutes_A ratio (Fig. S5 B and Data S1, table 19), which has been previously linked to lower BMI (Castaner et al., 2018).

Given the inherent batch effects between cohorts, when exploring differentially present species we prioritized those that were consistently elevated in cancer patients versus healthy donors across cohorts (Fig. 8 E and Data S1, tables 20 and 21). Consistent with the greater overall abundance of Bacteroidota, many species including the well-studied *Bacteroides fragilis* and *Bacteroides thetaiotaomicron* were more abundant in cancer patients (Fig. 8 F). In addition, several Firmicutes_A species, including many previously identified as potent regulatory T cell inducers (Atarashi et al., 2013; Narushima et al., 2014), particularly Clostridium_M bolteae, were also elevated in cancer patients versus healthy donors (Fig. S5, C and D). Finally, several species in the *Enterobacteriaceae* family, including the pathobionts *Escherichia coli_D*, *Escherichia dysenteriae*, and *Klebsiella pneumoniae*, were more prevalent in cancer patients across cohorts (Fig. 8 G and Data S1, tables 20 and 21). Previously observed as enriched in patients with IBD and colorectal cancer (Duvallet et al., 2017), *Enterobacteriaceae* are typically dominant in the upper GI tract and may become enriched with the faster stool transit time that occurs during diarrhea (Donaldson et al., 2016); without prior treatment and stool consistency information, we cannot tease apart if that is the cause for these patients.

In total, this analysis revealed that compared with their healthy counterparts, cancer patients’ gut microbiomes are less diverse and populated by more *Enterobacteriaceae*. However, counterintuitively, cancer patients also had higher Bacteroidota/Firmicutes_A ratios, which are associated with features of good health such as lower BMI, alanine aminotransferase, and C-reactive protein, as well as greater stability. While these observations are important for contextualizing findings of the recent cancer immunology/microbiome literature, future sampling efforts are needed to deconvolute which of these changes are driven by the cancer itself versus lifestyle changes or therapeutic agents that are taken after a cancer diagnosis and can themselves account for shifts in the gut microbiome. In addition, further experiments are needed to understand how these shifts may influence a patient’s underlying cancer immune set point and subsequent response to therapy (Chen and Mellman, 2017).

## Discussion

As the number of microbial intervention trials and biomarker studies continues to grow, it is increasingly important to develop a robust understanding of the gut microbiome across individuals in the steady state. In this study, we use the statistical power of a large cohort, the resolution afforded by deep shotgun sequencing, and an updated microbial database to expand our understanding of the gut microbiome in health and disease. More specifically, we identified sex as the strongest driver of community composition, with many *Prevotella* species enriched in men compared with women (Fig. 3 B); many of which were absent in previous databases (Truong et al., 2015) and thus not detectable in prior analyses. Given the recent literature on the strain-level variability within *Prevotella* species (De Filippis et al., 2019; Fehlner-Peach et al., 2019), particularly *Prevotella copri*, follow-up analyses should compare if there are also strain-level differences between the sexes.

Additionally, we identified 192 species associated with age (Fig. 4 B), greatly expanding what was known about the effects of aging on the gut microbiome (An et al., 2018). The changes seen here are particularly striking because they occur in the absence of underlying diseases or medication usage. Given the cross-sectional nature of this cohort, it is difficult to tease apart which of these associations is mediated by the variables correlated with age (Fig. S3 E), for example increased raw fruit, reduced fast food consumption, and increased BMI, in contrast to physiological changes associated with aging, such as thinning of the mucosal layer or altered pH levels. To definitively understand how the gut microbiome matures with aging will require longitudinal sampling of donors throughout their lifetimes, since in the short term, fluctuations are negligible in most donors (Fig. 6 A). Despite these caveats, the knowledge that bacterial communities are strongly shaped by age and sex encourages additional analysis into whether matching the age and sex of FMT donor and recipient could promote durable engraftment of bacteria.

These sex and age results could also have multiple implications for the interpretation of microbial biomarker studies. For example, several bacterial biomarkers have been reported for response to checkpoint inhibitors in non-GI cancer indications (Frankel et al., 2017; Gopalakrishnan et al., 2018; Matson et al., 2018; Peters et al., 2019; Routy et al., 2018). Notably, however, age—a prognostic biomarker of response in some indications and strong correlate of microbial composition—remains unaccounted for in some of those analyses. While our own attempts to associate PC1 and PC2 with response to therapy were unsuccessful (Fig. S5 E), these results should be interpreted with caution given the different definitions of response across cancer indications and interventional studies. Moving forward, statistically robust signatures of response will require larger cohort sizes and will benefit from analysis of continuous variables such as progression-free survival versus binary groupings (Peters et al., 2019). Additionally, while our observations concerning the differences between cancer patients and healthy donors should be carefully considered when designing FMT trials for this population, further experimental evidence is needed to determine whether the optimal donor is a patient who previously responded to therapy or a healthy donor with a responder-like signature.

In addition to cancer, these data will be valuable for designing microbial therapeutics for individuals of all ages. For instance, interventions containing *Bifidobacterium* species may need to be dosed more frequently in individuals older than 50 yr, in whom *Bifidobacterium* appears to colonize less effectively (Fig. 5, A, B, and D). Similarly, consortia with *Prevotella* species may work less effectively in females, and species which demonstrated low stability in the short term may also require additional dosing. Finally, beyond the findings in this paper, the rich metadata and 1,000-plus deep shotgun metagenomic samples provided here will be a valuable resource on which future microbiome studies can build new computational tools as well as generate and test new hypotheses.

## Materials and methods

### Experimental model and subject details

#### The MI cohort

The 1,000 healthy donors of the MI cohort were recruited by BioTrial in the suburban Rennes area (Ille-et-Vilaine, Bretagne, France). The cohort included 500 men and 500 women; 200 individuals were from each decade of life, between 20 and 69 yr of age. Participants were selected based on stringent inclusion and exclusion criteria, detailed elsewhere (Thomas et al., 2015). Donor BMI was restricted to ≥18.5 and ≤32 kg/m^2^. Briefly, the donors had no evidence of any severe/chronic/recurrent pathological conditions. Primary exclusion criteria were seropositivity for HIV or hepatitis C virus, travel to tropical or subtropical countries within the previous 6 mo, recent vaccine administration, and alcohol abuse. Subjects were also excluded if they took nasal, intestinal, or respiratory antibiotics or antiseptics any time in the 3 mo preceding enrollment. Additionally, anyone following a doctor- or dietician-prescribed diet for medical reasons (e.g., calorie-controlled diet in overweight patients) and volunteers with food intolerance or allergy were excluded. To avoid the influence of hormonal fluctuations in women during the perimenopausal phase, only pre- or postmenopausal women were included. To minimize the influence of population substructure, the study was restricted to individuals of self-reported metropolitan French origin for three generations (i.e., with parents and grandparents born in continental France).

#### Demographic, environmental, dietary, and clinical variables

Multiple demographic, environmental, and clinical variables were collected for each of the donors in an electronic case report form (Thomas et al., 2015). For example, donors were asked about their family medical history, smoking habits, sleeping habits, and infection and vaccination history. Additionally, donors completed a food-frequency questionnaire (FFQ) administered by trained investigators and comprising 19 food groups (Data S1, table 5). Participants estimated their “usual consumption” selecting from six intake frequencies ranging from “twice per day or more” to “never” (except for alcohol, which offered five intake frequencies ranging from “every day” to “never”). Investigators administering the FFQ invited participants to declare their “usual” diet, rather than focusing on their latest dietary consumption. The detailed FFQ is available in Partula et al. (2019). For clinical chemistry, hematologic, and serologic assessments, 20 ml of blood was collected from each donor and analyzed at the certified Laboratoire de Biologie Médicale, Centre Eugene Marquis (Rennes, France). For microbiome profiles, stool samples were produced by the participant at home within 24 h before the scheduled visits (visit 1 [V1] and V2). For individuals who provided two stool samples, V1 and V2 were on average 17 ± 3.3 d apart, minimum 8 d and maximum 45 d.

After manual curation and removal of variables that were (a) variable in <5% of participants, (b) missing in >25% of donors, or (c) correlated with another variable (Spearman ρ greater than −0.6 or < 0.6), 154 metadata variables were considered for future correlations. In the case of correlated variables (Data S1, tables 5 and 6), the variable with fewer missing values was prioritized and kept, while the other variable was removed. When the pair had equivalent numbers of missing values, one from the pair was randomly selected. Notably, circulating levels of creatinine were so strongly correlated with sex (Spearman ρ = 0.72, P = 3.5 × 10^−115^), this variable was excluded from the 154.

#### Ethics statement

The clinical study was approved by the Comité de Protection des Personnes–Ouest 6 on June 13, 2012, and by the French Agence Nationale de Sécurité du Médicament on June 22, 2012, and was performed in accordance with the Declaration of Helsinki. The study was sponsored by the Institut Pasteur (Pasteur ID-RCB no. 2012-A00238-35) and conducted as a single-center study without any investigational product. The original protocol is registered under ClinicalTrials.gov (study number NCT01699893). Informed consent was obtained from the participants after the nature and possible consequences of the studies were explained. The samples and data used in this study were formally established as the Milieu Interieur biocollection (NCT03905993), with approvals by the Comité de Protection des Personnes–Sud Méditerranée and the Commission nationale de l’informatique et des libertés on April 11, 2018.

### Details

#### Fecal DNA extraction and shotgun metagenomic sequencing

Stool specimens were collected in a double-lined sealable bag containing a GENbag Anaer atmosphere generator (Aerocult; Biomerieux) to maintain anaerobic conditions. Upon reception at the clinical site, fresh samples were aliquoted into cryotubes and stored at −80°C.

Stool aliquots were shipped to the CRO Diversigen for DNA extraction and shotgun metagenomic sequencing. At Diversigen, genomic DNA was extracted using PowerMag Soil DNA Isolation Kit (27100; Qiagen, MO BIO Laboratories). Libraries were prepared using Beckman robotic workstations (Biomek FX and FXp models) in batches of 96 samples. DNA (10–500 ng) was sheared into fragments of ∼300–400 bp in a Covaris E210 system (96-well format; Covaris) followed by purification of the fragmented DNA using AMPure XP beads. DNA end repair, 3′-adenylation, ligation to Illumina multiplexing PE adaptors, and ligation-mediated PCR were all completed using automated processes. To amplify high GC-rich and low AT-rich regions at greater efficiency, KAPA HiFi polymerase (KAPA Biosystems) was used for PCR amplification (6–10 cycles). Fragment Analyzer (Advanced Analytical Technologies) electrophoresis system was used for library quantification and size estimation. Prepared libraries were then pooled and sequenced on an Illumina HiSeq 2500.

In the end, we obtained 21 trillion raw paired-end reads from 1,359 samples from 946 of the donors. On average per sample, there were 2.4 Gbp, 15.5 million reads, with 358-bp insert size. To process the reads, Illumina TruSeq adapters were trimmed with Trimmomatic v0.36 (Bolger et al., 2014); low-quality and low-complexity reads were removed with prinseq-lite 0.20.4 (Schmieder and Edwards, 2011); and Bowtie2 v2.1.0 (Langmead and Salzberg, 2012) was used to remove reads mapping to PhiX or the PacBio human genome (parameters specified in Fig. S1 C). After processing, there were on average 13.9 ± 2.9 million reads per sample (Fig. S1, D and E). Of an initial 1,000 recruited donors, 44 were excluded from this analysis because of lack of consent for sharing their data outside of the MI consortium. An additional 10 donors were excluded because of technical issues in the extraction and sequencing steps (e.g., low DNA extraction yield), resulting in a sample size of 946 donors.

### Quantification and statistical analysis

#### Building the Kraken-GTDB database

To build the Kraken-GTDB database, first the following files were downloaded ftp://ftp.ncbi.nlm.nih.gov/genomes/refseq/bacteria/assembly_summary.txt and https://data.ace.uq.edu.au/public/gtdb/data/releases/release89/89.0/bac120_taxonomy_r89.tsv (Parks et al., 2018) on June 25, 2019. These files were merged based on accession number, and only those genomes present in both databases were considered, i.e., RefSeq genomes with a GTDB taxonomy. To avoid biasing the database toward those species with large numbers of genomes (Fig. S1 A), while balancing the added information provided by additional isolates per species, we selected up to five genomes per GTDB species to include in our database. Genomes were first ordered by their assembly quality, i.e., reference genome, representative genome, complete genome, chromosome, contig, and scaffold, and then randomly selected. Based on these criteria, 23,505 genomes representing 13,446 unique bacterial species were downloaded and formatted into a Kraken2 database (Wood and Salzberg, 2014). To incorporate the GTDB taxonomy into the Kraken2 database, files mimicking the NCBI-like taxonomy files from ftp://ftp.ncbi.nih.gov/pub/taxonomy/new_taxdump/new_taxdump.zip were created for names.dmp, complete_names.dmp, nodes.dmp, and accession2taxid. A matching Bracken database was then generated with bracken-build -k 35 -l 126 (Lu et al., 2017).

#### Metagenomic data analysis

First, putative reagent contaminants identified by species cocorrelation analysis were filtered using Kraken2’s *unclassified-out* option and a custom database of contaminant genomes (Data S1, table 22). Using our custom GTDB-Kraken database, Kraken2 v2.0.8 (Wood and Salzberg, 2014) and Bracken v2.5 (Lu et al., 2017) were run on the 1,359 quality-controlled samples (parameters specified in Fig. S1 F) to generate bacterial profiles. With the exception of longitudinal results in Fig. 6, all analyses were based on bacterial profiles from the V1 samples. In the case of eight donors from whom no V1 sample was available, the sample from V2 was used.

To complement results from the MI donors, this pipeline was run on an additional 1,159 shotgun metagenomics samples from 851 donors downloaded from ENA: PRJEB11532 (Zeevi et al., 2015), as well as 44 samples from 39 donors from SRP115355 (Frankel et al., 2017), 39 samples from 39 donors from SRP116709 (Matson et al., 2018), 25 samples from 25 donors from PRJEB22893 (Gopalakrishnan et al., 2018), 219 samples from 153 donors from PRJEB22863 (Routy et al., 2018), and 48 samples from 27 donors from PRJNA541981 (Fig. S4 A; Peters et al., 2019). Samples from PRJNA541981 had a large variability in sampling depths, 29 ± 31 million reads; therefore, 11 samples with >40 million reads were subsampled to 40 million reads. For the IBD comparison, an additional 220 samples from 220 donors were downloaded from the Lifelines and PRISM cohorts, PRJNA400072 (Franzosa et al., 2019), as well as 300 samples from 117 donors from iHMP, PRJNA389280 (Fig. S4 J; Schirmer et al., 2018). Donors in the iHMP <20 yr old were grouped in the pediatric cohort. For the Zeevi samples, metadata (sex, age, and BMI) was obtained by emailing the authors; no time point was provided, so an average of the microbial profiles across samples of the same donor were used in further analyses. For the oncology cohorts, one sample per donor was used for all but the longitudinal analysis. When available, the baseline sample was prioritized; otherwise an on-treatment sample was used. For the iHMP samples, an average of the microbial profiles across samples of the same donor were used.

To go beyond taxa-based calls, HUMANn2 v0.11.2 (Franzosa et al., 2018) with default parameters including Uniref90 was run on all MI samples where the forward and reverse reads were concatenated into a single file. Raw output values were converted from *rpks* to *cpms* with *humann2_renorm_table*. Outputs of all samples were joined into a single merged table with the *humann2_join_tables* function. Using *humann2_regroup_table*, individual gene families were regrouped with multiple different databases including COG, GO, KEGG, and MetaCyc. Ultimately, MetaCyc pathways (Caspi et al., 2018) were selected for the correlations because of the additional steps implemented in HUMANn2 to check for completeness of the pathways.

#### Statistical analysis

All correlations and statistical tests were performed in R v3.6.1 (R Core Team, 2019), documented via rmarkdown documents (Allaire et al., 2019), and compiled with knitr (Xie, 2019). Within R, tables were manipulated with functions of the dplyr package (Wickham et al., 2019). The majority of figures were rendered with ggplot2 (Wickham, 2016), adjusted with geasy (Carroll et al., 2020), and arranged with cowplot (Wilke, 2019). Colors were selected with the help of RColorBrewer (Neuwirth, 2014) and viridis (Garnier, 2018). The cladogram in Fig. 4 B was generated with GraPhlAn (Asnicar et al., 2015). Correlation plots in Fig. S3, D and E and Fig. 5 C were generated with ggcorrplot (Kassambara, 2019). Supplemental tables were generated with Openxlsx (Walker, 2019). When comparing values between two or more groups, Wilcoxon rank sum tests were used.

Bacterial α- and β-diversity measures including Shannon and BC were calculated using the R package vegan (Oksanen et al., 2019). To identify which of the 154 metadata variables were significantly associated with BC β-diversity, we used the adonis function in vegan to run PERMANOVA tests with 999 permutations (Oksanen et al., 2019). When repeated with 1,500, 2,000, 5,000, and 10,000 permutations, the same 52 variables were identified as significant (FDR ≤ 0.06). For all species-based analyses, we tested only those with prevalence >5% and a mean relative abundance >0.01% in the respective cohort. Similarly, for pathways, we prioritized those with prevalence >5%. To identify bacterial species differentially abundant between males/females and cancer patients/healthy donors, we input raw counts into DESeq2 (Love et al., 2014). Differences between sexes were adjusted for age and BMI, while differences between healthy and cancer were adjusted for age and sex. Because the latter led to the exclusion of samples from Matson et al. (2018), for which age and sex were not available, the comparison was also run without covariates (Data S1, table 21). Default values for the results function of DESeq were applied, including using Benjamini–Hochberg FDR for adjusting P values. To account for any lack of independence in the hypotheses being tested, multiple hypothesis correction using the Benjamini–Yekutieli adjustment was also performed and has been included in Data S1, tables 9, 10, and 21. Pathways differentially abundant between males and females were identified with Wilcoxon tests.

To identify bacterial species and pathways differentially prevalent between males and females, we used prop.test in R. Bacterial taxa and pathways associated with age were identified using Spearman correlations. To complement these analyses, GLM was also used. After performing arcsine square-root transformation of the relative abundances, models were fitted using the glm function in R, with sex and BMI included as covariates (model: species ~ age + sex + BMI; Data S1, table 12). For all correlations, species abundances were normalized with total sum scaling, and P values were adjusted with Benjamini–Hochberg. For conclusions based on species richness (e.g., Fig. 3 C and Fig. 5 B), we used linear models with sequencing depth as a covariate to validate that results were not an artifact of unequal library sizes.

To determine the stability of a donor’s bacterial species and pathways between V1 and V2, the BC distance was calculated and subtracted from 1. Individuals with a stability of 1 had samples that were identical across time points, while a stability of 0 meant the samples were nothing alike. Similarly, 1 − the Jaccard index was used to determine pathway stability based on presence/absence. To calculate between donor dissimilarities as shown in Fig. 6 A, for each individual we averaged the distance, as measured by BC, between that individual’s sample and all other individuals’ samples at V1. To identify the phyla associated with stability, Spearman correlation coefficients were calculated between the relative abundance of the phyla at baseline and stability as measured by 1 − BC. We then used GLM fit with the R package betareg v3.1-3 (Cribari-Neto and Zeileis, 2012) with a β response to identify which metadata factors were associated with intraindividual stability. To calculate stability of individual features (species and pathways), we again applied the BC metric, but this time compared the relative abundance of a single feature across all donors at V1 and V2.

To determine which continuous variables were associated with PC1, PC2, and Bacteroidota/Firmicutes_A ratios within the MI donors, we used Spearman correlations. When then applied linear models with age and sex as covariates to determine which of the associations was still significant after correcting for age and sex.

For all statistical tests, P values were corrected with the R function *p.adjust* using the Benjamini–Hochberg (FDR) method (Benjamini and Hochberg, 1995). To account for possible dependence between the hypotheses being tested across all species, multiple hypothesis correction using the Benjamini–Yekutieli adjustment was also performed, and those values have been included in Data S1, tables 9, 10, 12, 13, and 21 (Benjamini and Yekutieli, 2001).

### Data and software availability

#### Data availability

Sequence data have been deposited in the European Genome-Phenome Archive under accession code EGAS00001004437. Donor metadata and code used in this paper will also be available.

### Online supplemental material

Fig. S1 shows the quality control and Kraken analysis pipelines. Fig. S2 shows results from all the different program–database combinations tested. Fig. S3 shows distributions of the 154 metadata variables across the MI donors. Fig. S4 shows how cancer patients have altered gut bacterial profiles consistently across studies and indications. Fig. S5 shows additional analysis of which variables were associated with a cancer-like microbiome. 22 tables are provided in Data S1 that present summary statistics and other information.

## Supplementary Material

Data S1 contains 22 tables that present summary statistics and other information.
